# Aspirin Use and Survival Among Patients With Breast Cancer: A Systematic Review and Meta-Analysis

**DOI:** 10.1093/oncolo/oyad186

**Published:** 2023-06-26

**Authors:** Adam Baker, Christiana Kartsonaki

**Affiliations:** Department of Medical Sciences, Worcester College, University of Oxford, Oxford, UK; MRC Population Health Research Unit, Nuffield Department of Population Health, University of Oxford, Oxford, UK

**Keywords:** breast cancer, systematic review, meta-analysis, aspirin

## Abstract

**Background:**

Previous meta-analyses have indicated that aspirin could affect breast cancer outcomes, particularly when taken post-diagnostically. However, several recent studies appear to show little to no association between aspirin use and breast cancer mortality, all-cause mortality, or recurrence.

**Aims:**

This study aims to conduct an updated systematic review and meta-analysis on the associations of pre-diagnostic and post-diagnostic aspirin use with the aforementioned breast cancer outcomes. It also looks, through subgroup analyses and meta-regressions, at a range of variables that could explain the associations between aspirin use and breast cancer outcomes.

**Results:**

In total, 24 papers and 149 860 patients with breast cancer were included. Pre-diagnostic aspirin use was not associated with breast-cancer-specific mortality (HR 0.98, 95% CI, 0.80-1.20, *P* = .84) or recurrence (HR 0.94, 95% CI, 0.88-1.02, *P* = .13). Pre-diagnostic aspirin was associated with non-significantly higher all-cause mortality (HR 1.27, 95% CI, 0.95-1.72, *P* = .11). Post-diagnostic aspirin was not significantly associated with all-cause mortality (HR 0.87, 95% CI, 0.71-1.07, *P* = .18) or recurrence (HR 0.89, 95% CI, 0.67-1.16, *P* = .38). Post-diagnostic aspirin use was significantly associated with lower breast-cancer-specific mortality (HR 0.79, 95% CI, 0.64-0.98, *P* = .032).

**Conclusions:**

The only significant association of aspirin with breast cancer outcomes is lower breast-cancer-specific mortality in patients who used aspirin post-diagnostically. However, factors such as selection bias and high inter-study heterogeneity mean that this result should not be treated as conclusive, and more substantial evidence such as that provided by RCTs is needed before any decisions on new clinical uses for aspirin should be made.

## Teaser

This study conducted a detailed investigation into the associations between aspirin and mortality in patients with breast cancer.

**Implications for Practice**

Given the high worldwide prevalence of breast cancer, finding treatments able to reduce the proportion of women whose breast cancer is fatal by even a relatively small amount could lead to a large numerical reduction in breast cancer deaths. Previous meta-analyses of observational studies have indicated that aspirin could represent such a treatment. While our paper has similar findings, our analysis finds that there is good reason to be skeptical of aspirin’s association with reduced breast cancer fatality. Further evidence-gathering, such as the completion of ongoing RCTs, is needed to determine whether aspirin should be used in breast cancer treatment.

## Introduction

In 2020, there were an estimated 2.26 million new cases of female breast cancer and around 685 000 female breast cancer deaths worldwide.^1^ These figures make breast cancer both the most prevalent cancer in women and the cancer responsible for the greatest number of deaths in women. Finding new treatments for such a large contributor to global morbidity and mortality is a hugely desirable prospect.

Aspirin is a commonly used, inexpensive NSAID. Pre-clinical studies suggest a range of plausible mechanisms by which the effects of aspirin could improve survival in patients with breast cancer. One example is the inhibition of COX-2, overexpression of which has been associated with lower overall survival in patients with breast cancer.^2^ Another is the inhibition of platelet function, given that platelets are thought to contribute to cancer metastasis.^3^

The ADD-Aspirin trial^4^ is currently investigating the post-diagnostic use of aspirin in the treatment of breast cancer, but its primary completion date is estimated to be October 2026.^5^ A meta-analysis of observational evidence could therefore be useful until this point in assessing the potential value of aspirin as a treatment for breast cancer.

Multiple prior meta-analyses^6-9^ have found that aspirin, especially when taken after diagnosis of breast cancer, may be associated with reduced breast-cancer-specific mortality. However, 2 of these earlier meta-analyses were carried out a significant time ago, with literature searches up to 2014,^6,7^ whilst the more recent 2 did not distinguish between pre-diagnostic and post-diagnostic aspirin use.^8,9^ This study aims to build on these meta-analyses and conduct an updated and more detailed investigation into the associations between aspirin and mortality and recurrence in patients with breast cancer.

## Methods

### Search

We searched Ovid MEDLINE (including records from 1946 onwards) and Ovid EMBASE (including records from 1974 onwards), with publications up to and including June 30th, 2021 included. The full search strategy is shown in Appendices A and B. In addition to this, the reference lists of papers included in the secondary screening process were checked for relevant studies. After the primary screening process was completed, any papers identified as potentially relevant were screened independently by both authors, with differences resolved by discussion.

### Inclusion Criteria

RCTs, cohort studies, and case-control studies were eligible for inclusion, while reviews, meta-analyses and conference or poster abstracts with insufficient data were ineligible. Papers containing risk estimates and 95% CIs for breast-cancer-specific mortality, all-cause mortality, or recurrence were eligible for inclusion, as were those with sufficient data to calculate risk estimates. Only papers containing data on aspirin (and not NSAIDs as a whole or other NSAIDs) as the primary exposure were included.

### Data Extraction and Quality Assessment

AB extracted data from the selected papers, these data were then reviewed by CK. The categories of data extracted are given in Appendix C. We then performed a quality assessment using the nine-point Newcastle-Ottawa scale^10^ (NOS); studies scoring seven points or higher were considered high quality.

### Statistical Analysis

Analyses were carried out using R statistical software.^11,12^ When analyzing risk estimates, RRs and ORs were considered to be approximations of HRs, and all 3 measures were used interchangeably in common and random effects meta-analyses. Where ORs, RRs, or HRs were not given in the texts of studies, available data was used to calculate ORs or RRs. Where possible, RRs, ORs, or HRs which had been adjusted for confounders were used in meta-analyses, with unadjusted risk estimates only being included in analyses when papers did not provide adjusted estimates. Inter-study heterogeneity was assessed using the *I*^2^ statistic,^13^ with the heterogeneity of ≥50% considered substantial.^14^ Sensitivity analyses were used to assess sources of heterogeneity. Publication bias was evaluated using Egger’s test.^15^ Meta-regressions^16^ were used to assess the effect of individual variables on risk estimates. These variables were: year of publication, sample size, cancer stage at diagnosis, cancer grade at diagnosis, mean/median age, menopause status, smoking status, tumor molecular subtypes, cancer treatment, race, node-negative, or positive cancer at diagnosis, BMI, mean/median follow-up length, and the proportion of patients on low-dose or high-dose aspirin in a study (Supplementary Table S1).

## Results

### Characteristics of Included studies

**Figure 1.**
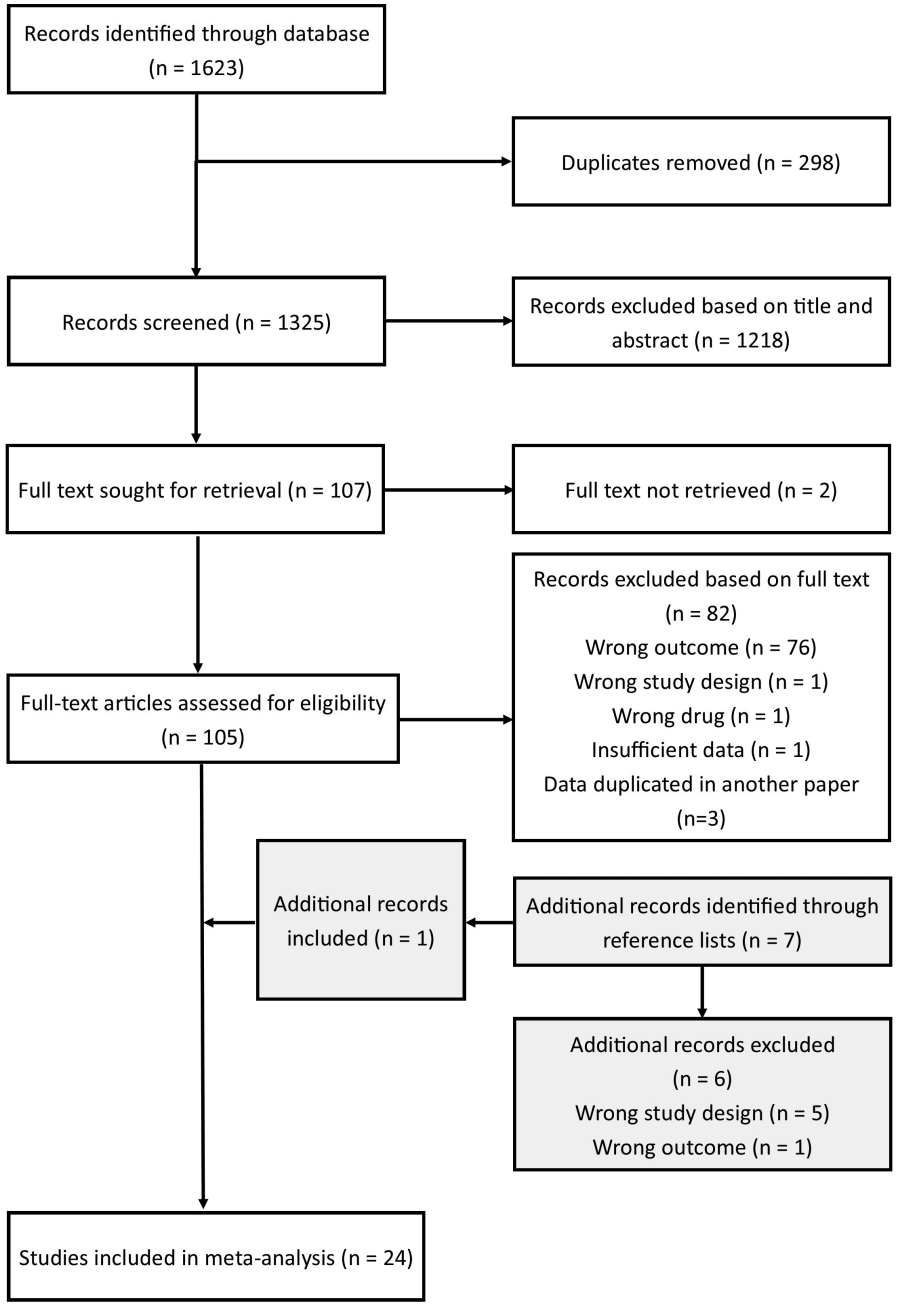
Study selection process.

A flowchart summarizing the search and study selection process is shown in Fig. 1. A total of 1325 papers identified through database searching were screened by title and abstract, with 1220 excluded due to irrelevance to this study, being a review or meta-analysis, or not having an available full-text version. A total of 105 studies had their full texts screened; 82 were excluded, with the majority of exclusions due to the paper’s primary outcome being breast cancer risk rather than an outcome relevant to this study. Screening the reference lists of these included studies yielded a further 7 potentially relevant studies, 1^17^ of which were eventually included in our meta-analysis. This meant a total of 24^17-40^ papers were chosen for the meta-analysis; 21 cohort studies^17-37^ and 3 case-control studies^38-40^ were included. Key characteristics of these papers are shown in Tables 1 and 2; confounders that they used to calculate adjusted risk estimates are listed in Supplementary Table S2. Altogether, 149 860 patients with breast cancer were enrolled in the studies in this meta-analysis. Supplementary Tables S3 and S4 show the methodological quality of the included studies; the mean NOS score was 7.8, indicating that included studies were generally of high quality.

**Table 1.** Key characteristics of included studies.

Citation details	Country	Design	Patients with breast cancer in study	Years of recruitment (for cohort study was based on)	Mean/median follow-up length	Period of aspirin exposure	Aspirin dose
Blair (2007)^18^	USA	Cohort study	591	1986-1992.	Median 8.3 years	Post-diagnostic	N/A
Kwan (2007)^19^	USA	Cohort study	2292	Cancer diagnosis between 1997 and 2000, aspirin use assessed 2000-2002	Mean 2.52 years	Pre- and post-diagnostic	N/A
Holmes (2010)^20^	USA	Cohort study	4164	1976-2002	N/A	Post-diagnostic	N/A
Wernli (2011)^21^	USA	Cohort study	3058	Breast cancer diagnosis 1988-1999, follow-up questionnaire 1998-2001.	Mean 7.2 years	Post-diagnostic	N/A
Rothwell (2012)^17^	UK	Cohort study using cohorts from 5 RCTs	81	BDAT 1978-1979, UK-TIA 1979-1985, TPT 1989-1992, POPADAD 1997-2001, AAA 1998-2001	Median 6.5 years	Pre- and post-diagnostic	Dose dependent on study—75 mg in TPT, 100 mg in POPADAD and AAA, 300 or 1200 mg in UK-TIA, 500 mg in BDAT
Li (2012)^22^	USA	Cohort study	1024	1996-2001.	N/A	“Lifetime” and “Recent” use assessed	N/A
Fraser (2014)^23^	Scotland (UK)	Cohort study	4627	January 1, 1993-December 31, 2008	Median 5.7 years	Pre- and post-diagnostic	99% of prescriptions for 75 mg
Barron (2014)^24^	Rep. of Ireland	Cohort study	2796	January 1, 2001-December 31, 2006	N/A	Pre- and post-diagnostic	85.4% of pre-diagnostic users exclusively used <150 mg/day. 85.7% of all prescriptions 75 mg, 10.8% 300 mg, 3.5% other dose
Barron (2015)^25^	Rep. of Ireland	Cohort study	4540	January 1, 2001-December 31, 2011	N/A	Post-diagnostic	95.5% of the cohort took <150 mg/day, 4.5% 150 mg
Cronin-Fenton (2016)^26^	Denmark	Cohort study	34 188	1996-2008	Median 7.1 years	Pre- and post-diagnostic	Low-dose
Bradley (2016)^27^	USA	Cohort study	2925	1993-2019 (recruitment for PLCO trial 1993-2001	N/A	Pre-diagnostic	N/A
Shiao (2017)^28^	USA	Cohort study	222	1998-2016	Median 41.3 months for the exposed group, 40.9 months for the controls	Post-diagnostic	N/A
McMenamin (2017)^29^	Scotland (UK)	Cohort study	15 140	January 2009-December 2012	Mean 4 years	Pre- and post-diagnostic	N/A
Strasser-Weippl (2018)^30^	Canada	Cohort study	2209	June 2, 2003-July 31, 2008	Median 4.1 years	Post-diagnostic	Low dose, max 81 mg
Frisk (2018)^31^	Sweden	Cohort study	22 035	April 1, 2006-December 31, 2012	Median 3.8 years	Pre- and post-diagnostic	75 mg or 160 mg
Wang (2018)^32^	USA	Cohort study	1442	1996-1997	Median 17.6 years	Pre-diagnostic	N/A
Williams (2018)^33^	USA	Cohort study	1113	2005-2013.	Mean 64.51 months	Pre- and post-diagnostic	N/A
Zhou (2019)^34^	USA	Cohort study	1227	2002-2013	Median 27 months for the 45 patients who underwent PIK3CA sequencing	Pre- and post-diagnostic	81 or 325 mg
Li (2020)^35^	USA	Cohort study	3152	January 1, 2016-December 31, 2016	Median 4.52 days for the aspirin group, 5.07 days for the non-aspirin group	Current, long-term use assessed	N/A
McCarthy (2020)^36^	USA	Cohort study	267	2009-2016	N/A	Post-diagnostic	51.9% 81 mg, 27.8% 325 mg, rest missing dose data
Loomans-Kropp (2021)^37^	USA	Cohort study	4552	November 8, 1993-July 2, 2001	N/A	Pre-diagnostic	N/A
Holmes (2014)^38^	Sweden	Nested case-control study	27 424	2005-2009	Median 2.57 years for all patients with breast cancer, 1.48 years for all deaths	Post-diagnostic	Low-dose (75 mg or 160 mg)
Sendur (2014)^39^	Turkey	Matched case-control study	974	2001-2013	Median 27.4 months	Pre-diagnostic	N/A
Murray (2014)^40^	UK	Nested case-control study	9817	1998-2007	Mean 6.9 years	Post-diagnostic (in the main analysis, pre-diagnostic compared in sensitivity analysis)	1.1% 25 mg, 97.1% 75 mg, 1.8% 300 mg

**Table 2.** Outcomes assessed in included studies.

Study	Pre BC death	Pre AC death	Pre Recurrence	Post BC death	Post AC death	Post recurrence
Blair (2007)				**X**	**X**	
Kwan (2007)			**X**			**X**
Holmes (2010)				**X**	**X**	**X**
Wernli (2011)				**X**	**X**	
Rothwell (2012)	**X**					
Li (2012)	**X**	**X**		**X**	**X**	
Fraser (2014)	**X**	**X**		**X**	**X**	
Barron (2014)	**X**	**X**				
Barron (2015)				**X**	**X**	
Cronin-Fenton (2016)			**X**			**X**
Bradley (2016)	**X**	**X**				
Shiao (2017)					**X**	**X**
McMenamin (2017)	**X**	**X**		**X**	**X**	
Strasser-Weippl (2018)					**X**	**X**
Frisk (2018)	**X**		**X**	**X**		
Wang (2018)	**X**	**X**				
Williams (2018)	**X**	**X**	**X**	**X**	**X**	**X**
Zhou (2019)		**X**	**X**		**X**	
Li (2020)					**X**	
McCarthy (2020)					**X**	
Loomans-Kropp (2021)	**X**					
Holmes (2014)				**X**	**X**	
Sendur (2014)		**X**	**X**			
Murray (2014)	**X**			**X**	**X**	

Abbreviations: pre, pre-diagnostic aspirin use; post, post-diagnostic aspirin use; BC death, breast-cancer-specific mortality; AC death, all-cause mortality; X, outcome assessed.

### Association of Pre-Diagnostic Aspirin Use With Breast Cancer Outcomes

#### Pre-Diagnostic Aspirin Use and Breast-Cancer-SpecificMortality

**Figure 2.**
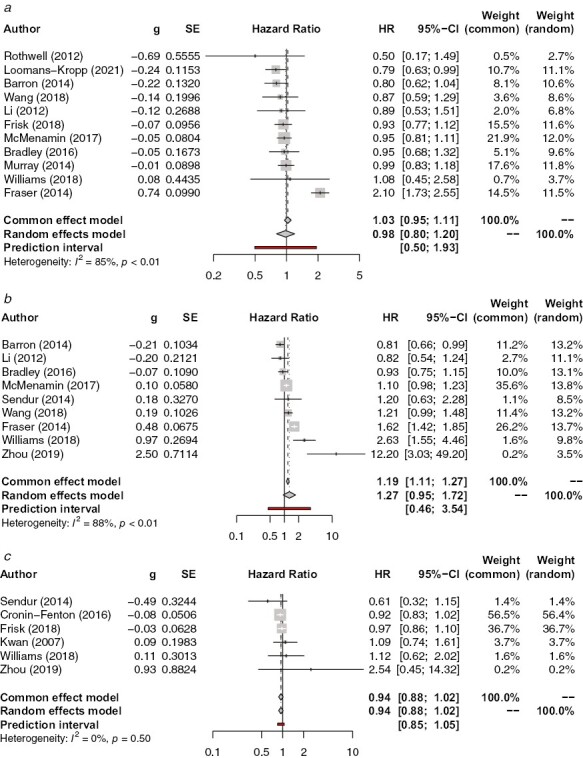
(**a**) Association between pre-diagnostic aspirin use and breast-cancer-specific mortality. (**b**) Association between pre-diagnostic aspirin use and all-cause mortality. (**c**) Association between pre-diagnostic aspirin use and recurrence.

There is no significant association of pre-diagnostic aspirin use with breast-cancer-specific mortality in either common (HR 1.03, 95% CI, 0.95-1.11, *P* = .47) or random (HR 0.98, 95% CI, 0.80-1.20, *P* = .84) effects models (Fig. 2a), as well as a large amount of heterogeneity (*I*^2^ = 85%). However, sensitivity analysis involving the removal of single papers to find sources of heterogeneity showed that the removal of Fraser et al.^23^ led to significantly decreased heterogeneity (*I*^2^ = 0%, 95% CI, 0%-62.4%). The risk estimate for breast-cancer-specific mortality also becomes significant when Fraser et al.’s paper is removed, albeit only barely (HR 0.91, 95%, CI 0.84-0.99, *P* = .022). Meta-analysis of additional data contained within 3 studies^27,31,32^ showed that patients who took less than 1 dose of aspirin per day had a similar likelihood of dying of breast cancer as those who took more than 1 dose of aspirin per day (HR = 0.90, 95% CI, 0.75-1.07, *P* = .24 for <1 daily dose and HR = 0.95, 95% CI, 0.75-1.19, *P* = .65 for >1 daily dose) (Supplementary Fig. S1). No evidence of publication bias in the papers used in this analysis was found using Egger’s test (*P* = .55) (Supplementary Fig. S2)

#### Pre-Diagnostic Aspirin Use and All-Cause Mortality

A common effects model indicates that all-cause mortality is significantly higher in patients who took aspirin pre-diagnostically (HR 1.19, 95% CI, 1.11-1.27, *P* < .0001), whilst a random effects model shows a larger, but non-significant increase (HR 1.27, 95% CI, 0.95-1.72, *P* = .11) (Fig. 2b). Heterogeneity is again high (*I*^2^ = 88%). Unlike breast-cancer-specificmortality, sensitivity analysis did not indicate that any one specific paper was responsible for this heterogeneity. Meta-regressions revealed multiple variables which were correlated with the magnitude of association. The proportion of patients treated with radiotherapy (*n* = 6, *P* < .0001) and the proportion of the cohort who were Black (*n* = 3, *P* = .0215) were both inversely correlated with an association of a greater magnitude. Meanwhile, the proportion of the cohort who were White (*n* = 4, *P* = .0054) and the proportion of the cohort who had never smoked (*n* =3, *P* = .0052) were both positively correlated with the magnitude of association (Supplementary Fig. S3). No evidence of publication bias was found (*P* = .66) (Supplementary Fig. S4).

#### Pre-Diagnostic Aspirin Use and Breast Cancer Recurrence

There is no significant association between pre-diagnostic aspirin use and breast cancer recurrence (HR 0.94, 95% CI, 0.88-1.02, *P* = .13) (Fig. 2c). There is very little heterogeneity (*I*^2^ = 0%, 95% CI, 0%-74.6%). No evidence of publication bias was found (*P* = .56) (Supplementary Fig. S5).

### Association of Post-Diagnostic Aspirin Use With Breast Cancer Outcomes

#### Post-Diagnostic Aspirin Use and Breast-Cancer-SpecificMortality

**Figure 3.**
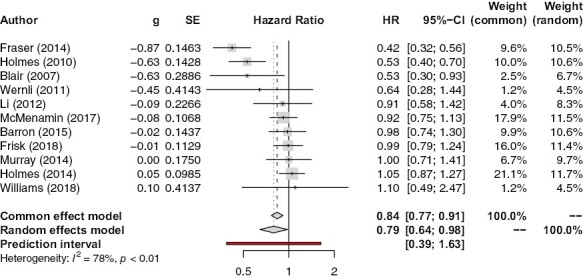
Association between post-diagnostic aspirin use and breast-cancer-specific mortality.

Fig. 3 shows the association between post-diagnostic aspirin use and breast-cancer-specific mortality. In both common (HR 0.84, 95% CI, 0.77-0.91, *P* < .0001) and random (HR 0.79, 95% CI, 0.64-0.98, *P* = .032) effects models, there is statistically significantly lower breast cancer mortality. Heterogeneity is large (*I*^2^ = 78%): removing Fraser et al.^23^ again somewhat reduces heterogeneity (*I*^2^ = 59%), as well as the magnitude of association (HR 0.90, 95% CI, 0.82-0.99, *P* = .026 in a common effects model and HR 0.86, 95% CI, 0.73-1.02, *P* = .09 in a random effects model). When all studies which assessed aspirin use through self-reporting^18,20,21^ were removed from the analysis, the magnitude of the association between aspirin and breast cancer death became less extreme in both common (HR = 0.90, 95% CI, 0.82-0.99, *P* = .026) and random (HR = 0.88, 95% CI = 0.70-1.10, *P* = .26) effects models, although heterogeneity remained high (*I*^2^ = 78%). Removing Fraser et al. ^23^ in addition to these 3 papers led to a large reduction in heterogeneity (*I*^2^ = 0%, 95% CI, 0%-70.8%), and to there no longer being a significant association between post-diagnostic aspirin use and breast-cancer-specific mortality (HR = 0.99, 95% CI, 0.89-1.09, *P* = .78).

Meta-analysis of additional data from 4 studies^20,29,31,38^ showed that post-diagnostic users of aspirin with stage I cancer at diagnosis (HR = 0.56 in both common and random effects models, 95% CI, 0.40-0.79, *P* = .0011 in common effects model and 95% CI, 0.34-0.91, *P* = .02 in random effects model) had lower breast-cancer-specific mortality than the post-diagnostic users of aspirin with stage II cancer at diagnosis (HR = 0.92, 95% CI, 0.78-1.09, *P* = .35 in common effects model, HR = 0.83, 95% CI, 0.53-1.29, *P* = .41 in random effects model) and that post-diagnostic users of aspirin with stage III cancer at diagnosis had a non-significant increase in breast-cancer-specific death (HR = 1.07, 95% CI, 0.82-1.39, *P* = .64 in common effects model, HR = 1.05, 95%, CI 0.62-1.76, *P* = .86 in random effects model). Another meta-analysis of data from 3 studies^29,31,38^ found that the association between post-diagnostic aspirin use and reduced breast-cancer-specific death was slightly stronger in patients with ER-positive cancer than in the whole cohort (HR = 0.79, 95% CI, 0.66-0.95, *P* = .013 in common effects model and HR = 0.71, 95% CI, 0.46-1.11, *P* = .13 in random effects model). All of these results are shown in Supplementary Fig. S6.

**Figure 4.**
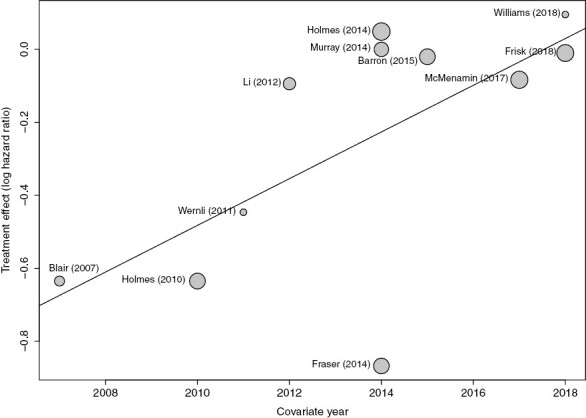
Bubble plot showing correlation between study year and association of post-diagnostic aspirin with breast-cancer-specific mortality.

Meta-regression revealed that later study year was associated with a reduced magnitude of association between post-diagnostic aspirin use and breast-cancer-specific death (*n* = 11, *P* = .0419) (Fig. 4). Another meta-regression showed that the percentage of patients in a study’s cohort with node-positive cancer at diagnosis was inversely correlated with magnitude of association (*n* = 4, *P* = .0002) (Supplementary Fig. S7).

No evidence of publication bias (*P* = .44) was found in the papers used in this analysis (Supplementary Fig. S8).

#### Post-Diagnostic Aspirin Use and All-Cause Mortality

**Figure 5.**
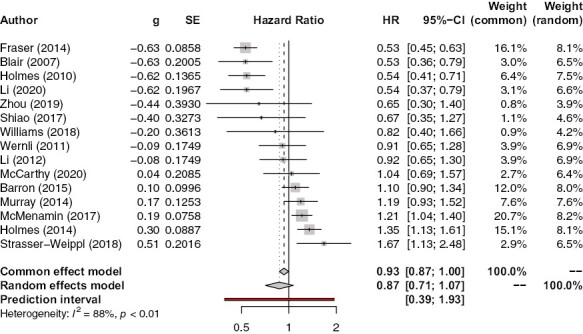
Association between post-diagnostic aspirin use and all-cause mortality.

Fig. 5 shows the association between post-diagnostic aspirin use and all-cause mortality. There are small reductions in all-cause mortality in both common (HR 0.93, 95% CI, 0.87-1.00, *P* = .041) and random (HR 0.87, 95% CI, 0.71-1.07, *P* = .18) effects models, in addition to a large amount of heterogeneity (*I*^2^ = 88%). Meta-regression revealed that the proportion of a paper’s cohort who were black was positively correlated with an increased magnitude of association between post-diagnostic aspirin and all-cause death (*n* = 4, *P* = .0026) (Supplementary Fig. S9). There was no evidence of publication bias (*P* = .44) (Supplementary Fig. S10).

#### Post-Diagnostic Aspirin Use and Breast Cancer Recurrence

**Figure 6.**
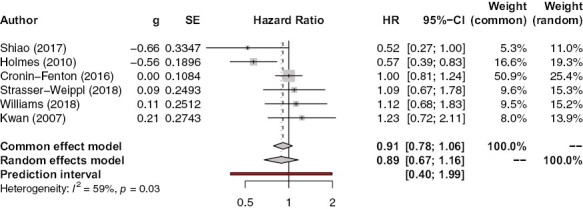
Association between post-diagnostic aspirin use and recurrence.

Fig. 6 shows the association between post-diagnostic aspirin use and breast cancer recurrence. Similarly to the results on the association of pre-diagnostic aspirin use with recurrence, there is no significant association between post-diagnostic aspirin use and breast cancer recurrence in both common (HR 0.91, 95% CI, 0.78-1.06, *P* = .23) and random (HR 0.89, 95% CI, 0.67-1.16, *P* = .38) effects models. There is a fairly substantial amount of heterogeneity (*I*^2^ = 59%); sensitivity analysis showed that the removal of Holmes et al.’s paper^20^ reduced both heterogeneity (*I*^2^ = 15.5%) and effect size (HR= 1.00, 95% CI, = 0.85-1.18, *P* = .99).

There was no evidence of publication bias (*P* = .71) (Supplementary Fig. S11).

## Discussion

This meta-analysis looked at the association between pre-diagnostic or post-diagnostic aspirin use and breast cancer outcomes. It included 24 studies with a pooled cohort size of 149 860 patients with breast cancer. The study showed that pre-diagnostic aspirin use was not significantly associated with breast-cancer-specific mortality or recurrence, and appeared to be associated with slightly higher all-cause mortality. However, this latter finding is perhaps not unexpected given that a major reason for long-term aspirin use is in the secondary prevention of cardiovascular events,^41^ and therefore patients taking aspirin could be expected to have a higher likelihood of poor health irrespective of breast cancer diagnosis. Meta-regression revealed a number of variables associated with the magnitude of association between pre-diagnostic aspirin and all-cause mortality, including race, smoking status, and the proportion of patients in a cohort treated with radiotherapy. The proportion of a cohort who were Black being correlated with a reduced association between pre-diagnostic aspirin and all-cause mortality could potentially be explained by the fact that Black people have a higher overall mortality rate compared to White people in the USA,^42^ where many studies included in this analysis were conducted, and by the fact that Black people have breast cancer features associated with worse prognoses more commonly than other races.^43^ Additionally, as smoking is linked to increased incidence of CVD,^44^ it can be inferred that a greater proportion of smokers than non-smokers would be taking aspirin due to its use as a secondary preventative measure in patients with CVD. This, combined with the overall higher mortality rates in smokers,^45^ could explain the reduced association between pre-diagnostic aspirin and higher all-cause death in cohorts with more never smokers. Finally, a greater proportion of patients in a cohort who were treated with radiotherapy being associated with reduced magnitude of association between aspirin and all-cause mortality could potentially be explained by an interaction between radiation-associated CVD^46^ and patients taking aspirin pre-diagnostically as a result of existing poor cardiovascular health.

It should be noted that the small number of papers used in these meta-regressions limits the statistical power of these results. This is illustrated by the fact that the removal or inclusion of single papers was able to determine whether or not meta-regressions of other variables were statistically significant. For example, meta-regressions found that the proportion of a cohort’s patients with stage IV cancer was significantly correlated with the magnitude of association between pre- or post-diagnostic aspirin and all-cause and breast cancer mortality, but in all 4 cases this finding was rendered non-significant when Fraser et al.^23^ was removed from the analysis. In addition, the proportion of a cohort’s patients with grade 2 cancer was significantly correlated with pre-diagnostic aspirin’s magnitude of association with all-cause mortality only when Zhou et al. ^34^ was excluded from the meta-regression, but the proportion of a cohort’s patients treated with chemotherapy was significantly associated with the same outcome only when Zhou et al.’s paper was included.

These 2 papers likely had such a disproportionate effect on whether or not a variable was significantly correlated with magnitudes of association between aspirin use and breast cancer outcomes because they themselves found relatively strong associations between aspirin use and some outcomes, thus causing correspondingly large changes in the magnitude of association between said variables and breast cancer outcomes with their removal or inclusion from meta-regression calculations, especially when few other studies were involved in said calculations.

Our study also found that post-diagnostic aspirin use was not significantly associated with all-cause mortality or recurrence, but that post-diagnostic aspirin use appeared to be somewhat associated with breast cancer mortality. There are multiple potential mechanisms for this association. One hypothesis is that aspirin’s inhibition of COX-2 leads to a beneficial effect, given that COX-2 overexpression has been linked to worse overall survival^2^ in breast cancer. COX activity could lead to worse cancer outcomes through multiple pathways, such as promotion of the epithelial-to-mesenchymal transition^47^ and induction of angiogenesis,^48^ so inhibiting COX could improve breast cancer survival. Another potential mechanism by which aspirin could improve breast cancer survival is a reduction in cancer metastasis through inhibition of platelet function.^3,49,50^

The fact that post-diagnostic aspirin use was found to be significantly associated with lower breast-cancer-specific mortality, despite not being found to be significantly associated with lower disease recurrence, could be due to a smaller number of papers including data on the association between post-diagnostic aspirin use and breast cancer recurrence, with this smaller dataset making it less likely for any association to reach statistical significance. It could also reflect the fact that only 1^20^ of the 4^18,20,21,23^ papers with the strongest association between post-diagnostic aspirin use and breast-cancer-specific mortality included data on the association between post-diagnostic aspirin use and breast cancer recurrence.

The magnitude of the association between post-diagnostic aspirin and breast-cancer-specific mortality which this study finds is smaller than those seen in 3 previous meta-analyses,^6,7,9^ although it is similar to that found in a more recent meta-analysis.^8^ As 2 previous meta-analyses were published in 2015, the difference between their findings and that of this paper matches the correlation between later study years and a reduced magnitude of association between post-diagnostic aspirin use and breast-cancer-specific mortality which we observed through meta-regression. Possible explanations for this correlation include the fact that papers published later tended to have a lower follow-up length, or the fact that the 3 papers^18,20,21^ which used self-reported aspirin use data were the 3 earliest papers to look at the association between post-diagnostic aspirin and breast cancer mortality. Additionally, another relatively early paper with positive results^23^ may have had these results influenced by its method of ascertaining aspirin use; as post-diagnostic aspirin use was assessed based on prescriptions up to the time of death, patients not having their prescriptions renewed when close to death^51^ could have implied an erroneously strong association between aspirin use and breast cancer mortality. Our finding that post-diagnostic aspirin use in patients with ER-positive breast cancer was associated with a slightly stronger association with breast-cancer-specific mortality than in the study cohort as a whole is supported by a previous finding^52^ that NSAID use was associated with reduced breast-cancer-specific mortality in patients with ER-positive cancer, and with increased breast-cancer-specific mortality in patients with ER-negative cancer. A possible explanation for this effect could be the finding that NSAIDs such as aspirin can reduce serum estrogen,^53^ which could potentially reduce the growth of estrogen-responsive tumors. Finally, we also found that post-diagnostic aspirin use was more strongly associated with reduced breast-cancer-specific mortality in patients with lower-stage cancer at diagnosis. This suggests that aspirin may exert most of its effects in small, local cancers, perhaps through inhibiting tumor growth^54^ and thus reducing the ability of the primary tumor to seed metastases.^55,56^

Our study’s finding that pre-diagnostic aspirin use is not significantly associated with breast cancer outcomes is also seen in both prior meta-analyses^6,7^ which looked specifically at pre-diagnostic or post-diagnostic aspirin use. These 2 meta-analyses also support this study’s finding that post-diagnostic aspirin use is associated with a non-significant decrease in all-cause mortality. Only 2 prior meta-analyses^7,9^ looked at the association between aspirin use and recurrence; one^7^ finds that post-diagnostic aspirin use causes a larger decrease in recurrence than was observed by our study, whilst the second^9^ finds similar results to this study. Conversely, a recent RCT, the ABC trial,^57^ assessing whether post-diagnostic aspirin use can prevent recurrence in breast cancer found a small increase in recurrences in the treatment arm of the study compared to the control arm.^58^ Overall, the results of our study appear similar to those from prior meta-analyses which looked at this subject, with the exception of a slightly smaller magnitude of the association being found between post-diagnostic aspirin use and breast-cancer-specific mortality.

Strengths of this meta-analysis include that it separates all results by whether aspirin was taken before or after diagnosis, that it includes subgroup analyses of variables that are not included in similar meta-analyses, and that it contains a number of meta-regressions that could help explain aspirin’s associations with various breast cancer outcomes.

However, there are numerous limitations to this meta-analysis, not least the fact that the data that it includes is observational. First, the sample size for some meta-analyses is somewhat small, especially the analyses of aspirin’s associations with recurrence. In addition to this, not all studies had data for all variables which were looked at via meta-regression, so some meta-regressions were also limited by small sample sizes; this may explain why meta-regressions looking at stage or ER status did not show significant correlations, contrary to this study’s meta-analyses which indicated that these variables affected aspirin’s associations with breast cancer outcomes. In most analyses there tended to be a large amount of heterogeneity, which could be explained by a number of factors; one could be the large range of study follow-up lengths, whilst another could be the differences in aspirin use assessment between studies. Finally, it should be noted that all studies included in this meta-analysis were performed in wealthy nations in Europe or North America, with one study performed in Turkey^39^ a potential exception. Caution must therefore be used in extrapolating these results to less wealthy nations where the standards of cancer treatment are often markedly different. The same point could be made about ethnicity, given that most countries in this study have majority White populations and different ethnicities are known to have different cancer characteristics and outcomes.^43^

Our study finds that aspirin taken pre-diagnostically has little association with breast cancer outcomes. However, given that other meta-analyses^59^ have found that pre-diagnostic aspirin use is associated with a reduced risk of a patient developing breast cancer, aspirin could still be a useful prophylactic option in populations at a high risk of developing breast cancer, such as those with a family history of the disease. It also finds that aspirin taken post-diagnostically may have a limited beneficial association with breast cancer mortality. Caution must be used when interpreting this result as the apparent significant association with breast-cancer-specific mortality is largely generated by 3 papers^18,20,21^ which use self-reported aspirin usage data, and another paper^23^ whose positive results may have at least partially been due to reverse causation. Given this, it may be prudent to wait for the results of the ADD-Aspirin trial^4^ to draw firm conclusions about post-diagnostic aspirin use’s association with breast cancer mortality. Aside from the fact that RCTs are generally viewed as a higher standard of evidence than observational studies, ADD-Aspirin has an arm consisting of patients taking 300mg of aspirin daily. Given that many of the studies in this meta-analysis involved patients taking low-dose aspirin, it could be that this arm of ADD-Aspirin generates a different result than this study found.

Should ADD-Aspirin, or other future RCTs, confirm this study’s finding that post-diagnostic aspirin has some beneficial association with breast cancer mortality, then aspirin could be used as a long-term therapy in patients with breast cancer after primary treatment. Due to its low cost, aspirin could be especially useful in lower-income countries where more expensive breast cancer treatments are less readily available. Aside from its potential clinical uses, knowledge of the effects of aspirin on breast cancer outcomes could allow for its use to be identified as a confounding variable in other breast cancer studies.

Future studies could look for cohorts in whom aspirin is especially beneficial to allow its use to be more selectively indicated. They could look at variables such as ER status or stage at diagnosis, which were identified in this meta-analysis. Another, less well-studied variable that could be looked at is COX status. Given that aspirin is known to have effects on COX enzymes, it is surprising that there are few studies that analyze the effect of COX status on aspirin’s association with breast cancer outcomes. Whilst it should be noted that one study^60^ which looked at the relationship between COX status and aspirin’s association with breast cancer outcomes found little difference between patients with COX positive or negative tumors, this study looked only at COX-2 status, so future studies could instead examine COX-1 status. Additionally, it could be useful for future studies analyzing the effects of aspirin on breast cancer outcomes to look at populations outside of Europe or North America, given that all of the studies in this meta-analysis were conducted in those regions. The ADD-Aspirin trial is already doing this, as it recruited a portion of its participants from India.

## Supplementary Material

oyad186_suppl_Supplementary_Figures_1-11

oyad186_suppl_Supplementary_Table_1

oyad186_suppl_Supplementary_Tables_2-4

## Data Availability

The datasets generated during and/or analyzed during the current study are available in the following Github Repository: https://github.com/abaker2001/Aspirin-Systematic-Review-Code

## Registration and Protocol

The registration and protocol for this study can be accessed via the following link: https://www.crd.york.ac.uk/prospero/display_record.php?RecordID=259906

## Appendices

### Appendix A—MEDLINE search terms

1. exp Breast Neoplasms/
2. ((breast) adj5 (cancer* or tumour* or tumor* or neoplas* or malignan* or carcinoma* or adenocarcinoma*)).mp.
3. 1 or 2
4. (aspirin or acetylsalicylic acid).mp.
5. 3 and 4
6. Limit 5 to humans
7. Review.pt.
8. 6 not 7

### Appendix B—EMBASE search terms

EMBASE Ovid

1. exp breast cancer/
2. ((breast) adj5 (cancer* or tumour* or tumor* or neoplas* or malignan* or carcinoma* or adenocarcinoma*)).mp.
3. 1 OR 2
4. (aspirin or acetylsalicylic acid).mp.
5. 3 and 4
6. Conference.pt.
7. 5 NOT 6
8. Limit 7 to human
9. Review.pt.
10. 8 not 9

### Appendix C—Categories of extracted data

1. Citation details (paper title, authors, year of publication, and journal name, volume, issue, and page number/s)
2. Country/countries of study population
3. Study design (eg, cohort or case-control, prospective or retrospective, etc.)
4. Total number of participants in study cohort
5. Size of control and exposed (to aspirin) groups in cohort studies, or number of cases and controls in case-control studies
6. Characteristics of study cohort (including age, sex, race, weight, smoking status, menopause status, cancer treatment, stage of disease, node status of disease, metastasis status of disease, grade of disease, molecular subtype of disease [eg, HER2 or oestrogen receptor status])
7. Years of participant recruitment
8. Duration of follow-up and mean or median follow-up period
9. Details of exposure to aspirin in group (including whether exposure was pre- or post-diagnostic, or both, and the dose or doses of aspirin taken by study participants)
10. Details on breast cancer mortality in pre- and post-diagnostic aspirin users and controls (including the number of deaths from breast cancer in users and controls, unadjusted and adjusted risk estimates with 95% CIs, and data stratified by stage of disease, menopause status, molecular subtype of disease, BMI, other cancer treatment and aspirin dose)
11. Details on all-cause mortality in pre- and post-diagnostic aspirin users and controls (including the number of all-cause deaths in users and controls, unadjusted and adjusted risk estimates with 95% CIs, and data stratified by factors such as those established in point [10])
12. Details on recurrence in pre- and post-diagnostic aspirin users and controls (including the number of recurrences or disease-free survival events in users and controls, unadjusted and adjusted risk estimates with 95% CIs, and data stratified by factors such as those established in point [10])
13. Details on progression of disease in pre- and post-diagnostic aspirin users and controls (including numbers of patients with local and metastatic disease in the aspirin use and control groups, unadjusted and adjusted risk estimates with 95% CIs and data stratified by factors such as those established in point [10])
14. Confounding variables assessed in multivariate analysis
