# Supplementary material for: Aspirin Use and Survival Among Patients With Breast Cancer: A Systematic Review and Meta-Analysis
Source: Oncologist. 2023 Jun 26;29(1):e1–e14. doi: 10.1093/oncolo/oyad186 (PMC10769789; doi:10.1093/oncolo/oyad186)

***Supplementary Figure 1a- Forest plot showing association between less than one pre-diagnostic daily dose of aspirin and breast cancer-specific death***

***
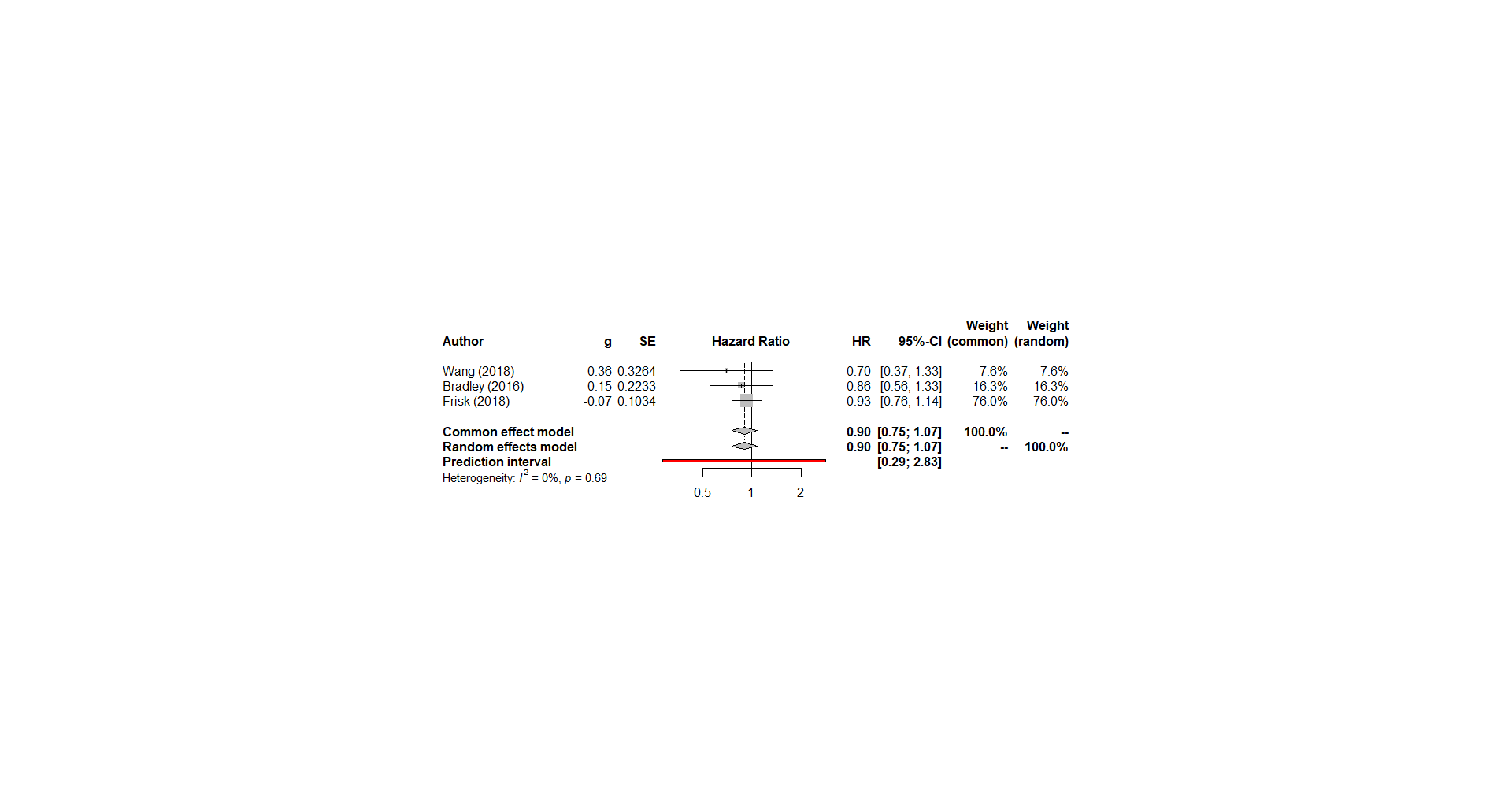
***

***Supplementary Figure 1b- Forest plot showing association between more than one pre-diagnostic daily dose of aspirin and breast cancer-specific death***

***
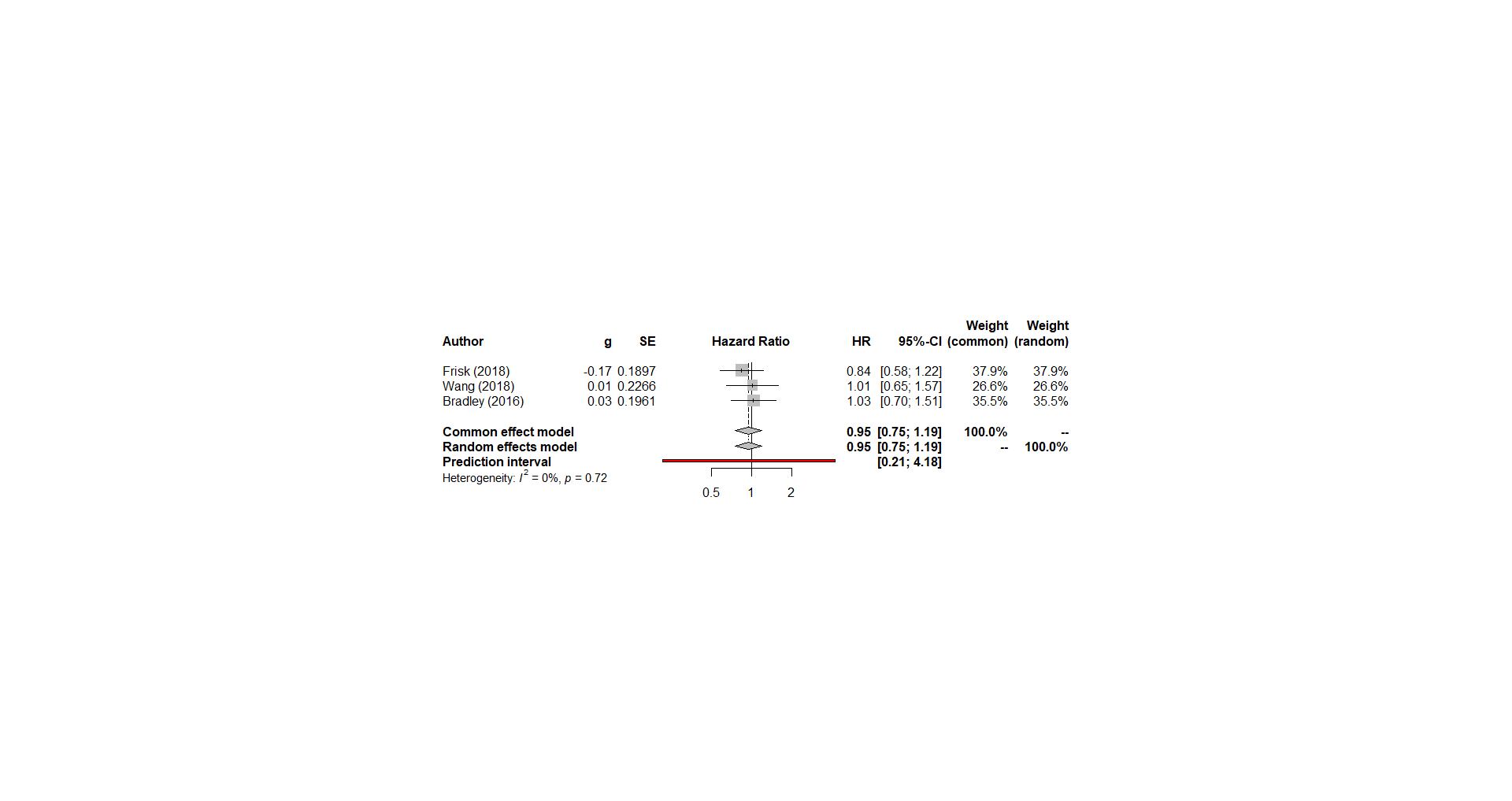
***

***Supplementary Figure 2- Funnel plot of studies included in the analysis of pre-diagnostic aspirin use and breast cancer-specific mortality***


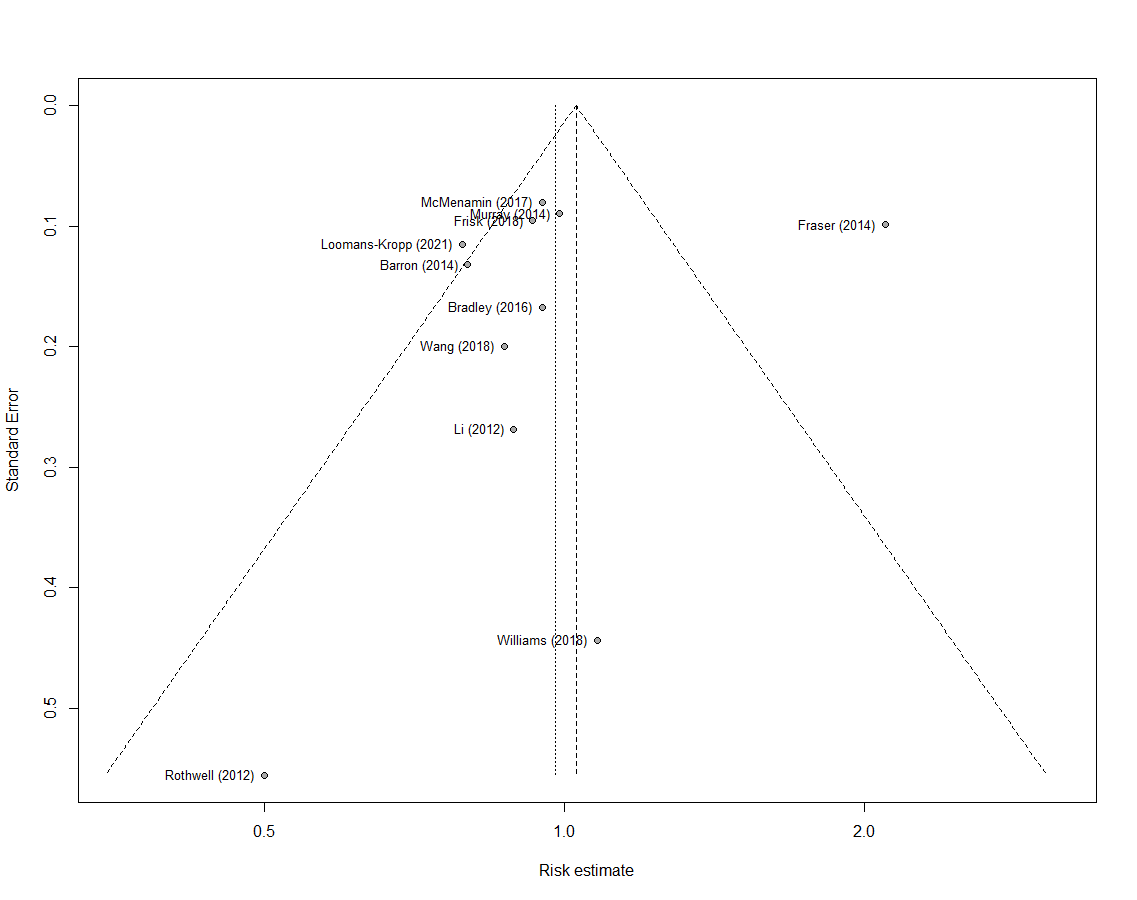


***Supplementary Figure 3a- Bubble plot comparing association between pre-diagnostic aspirin and all-cause death and proportion of each cohort treated with radiotherapy***


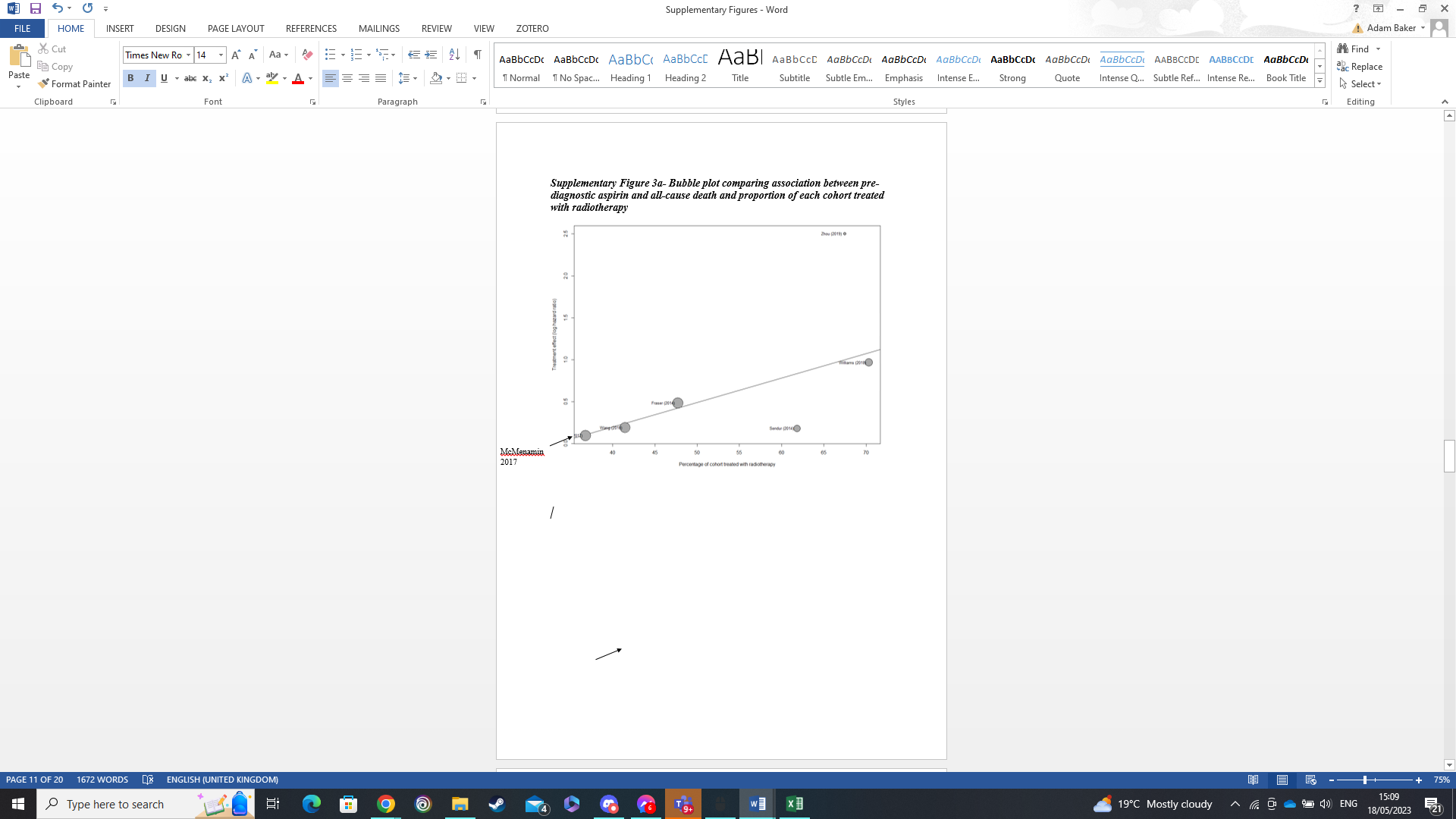


***Supplementary Figure 3b- Bubble plot comparing association between pre-diagnostic aspirin and all-cause death and proportion of each cohort who were White***


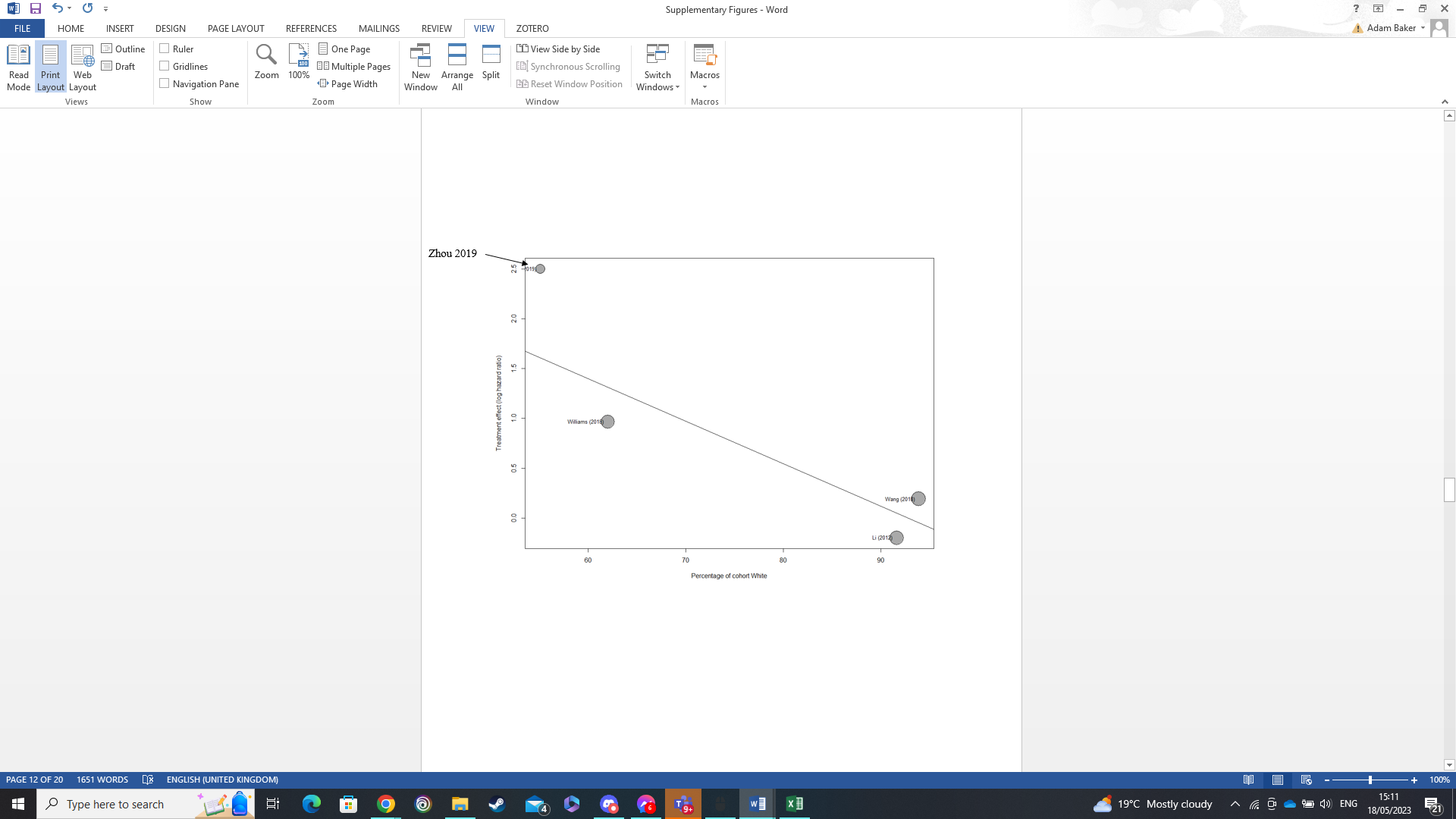


***Supplementary Figure 3c- Bubble plot comparing association between pre-diagnostic aspirin and all-cause death and proportion of each cohort who were Black***


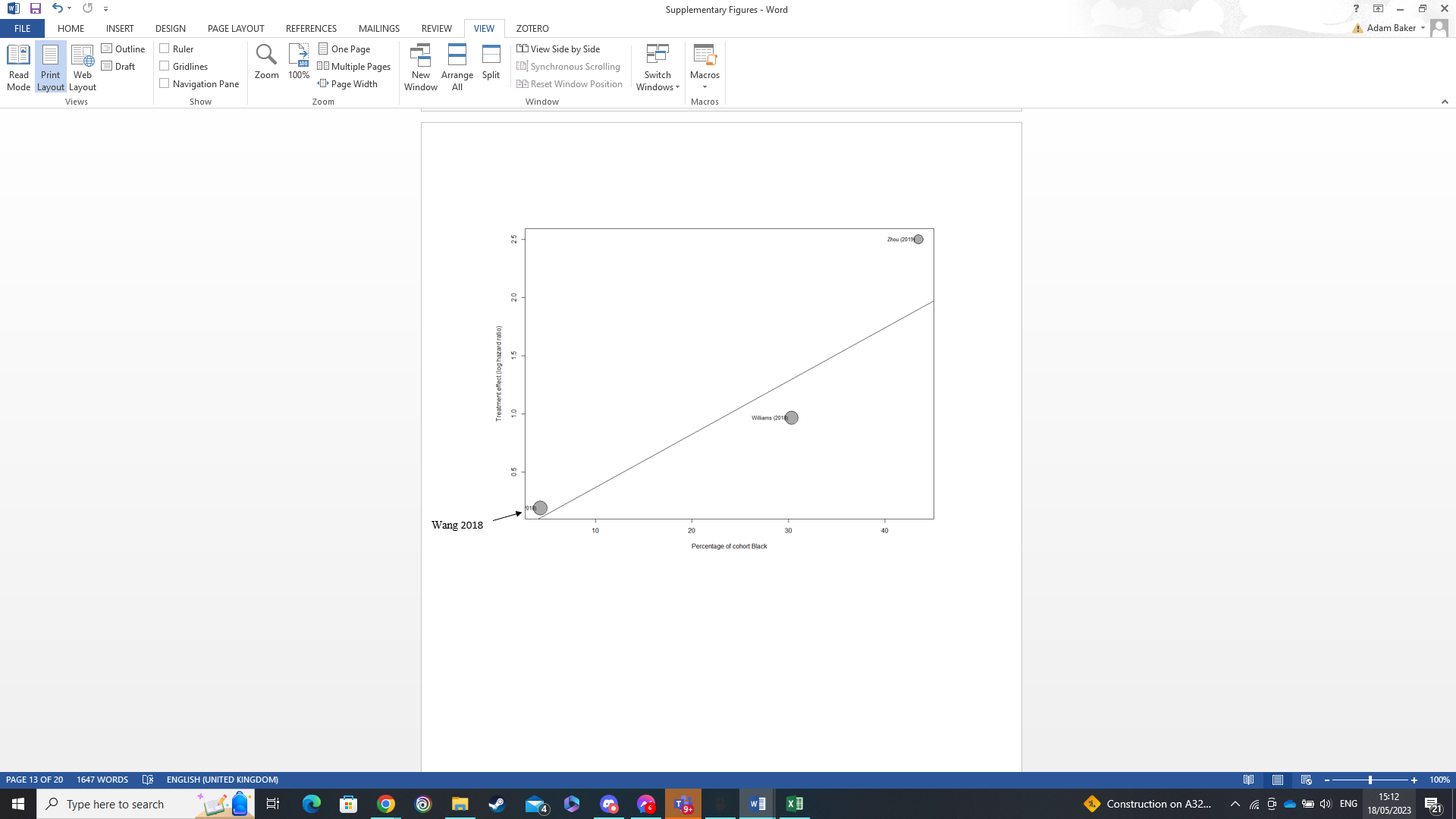


***Supplementary Figure 3d- Bubble plot comparing association between pre-diagnostic aspirin and all-cause death and proportion of each cohort who had never smoked***


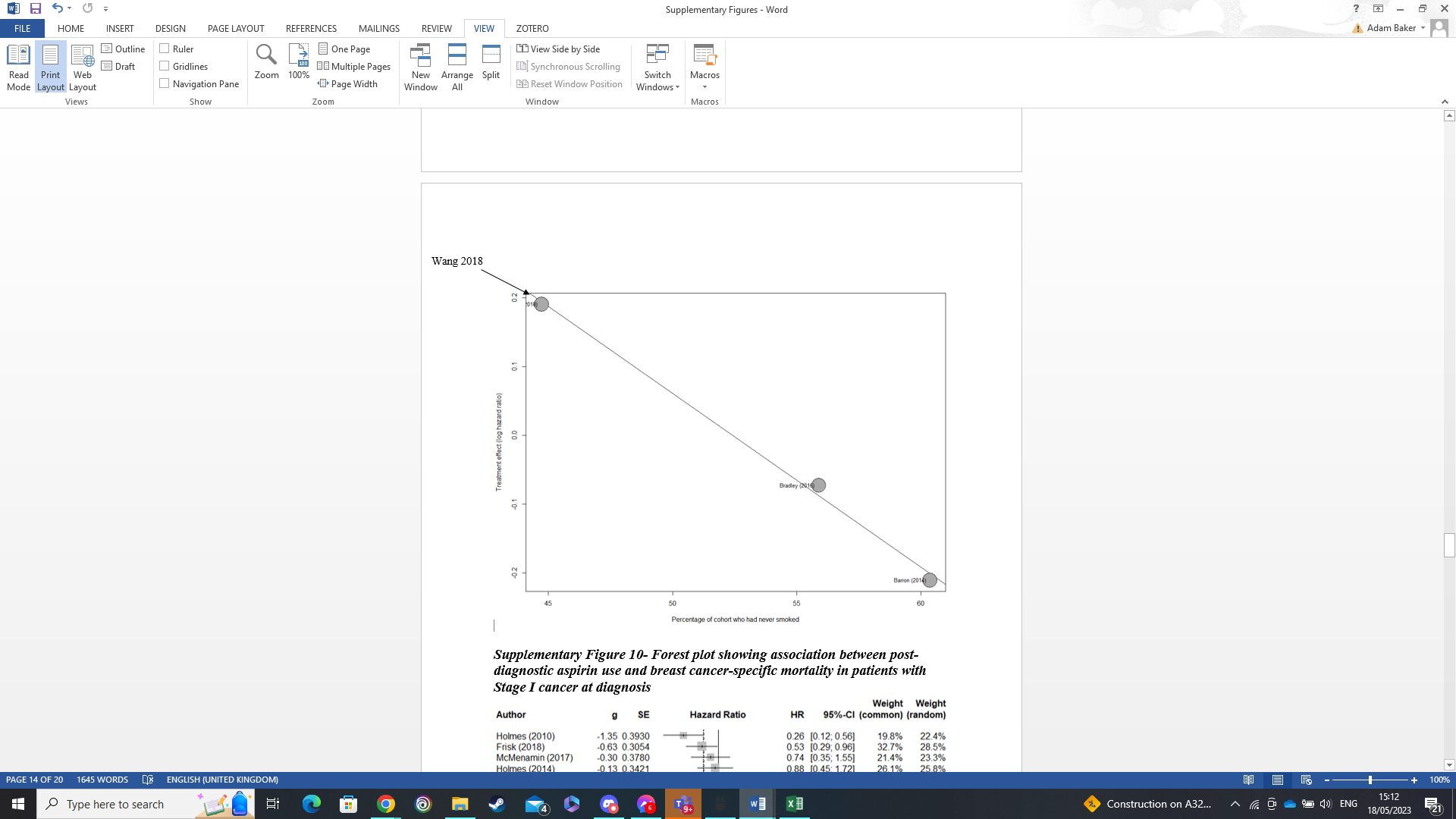


***Supplementary Figure 4- Funnel plot of studies included in the analysis of pre-diagnostic aspirin use and all-cause mortality***

***
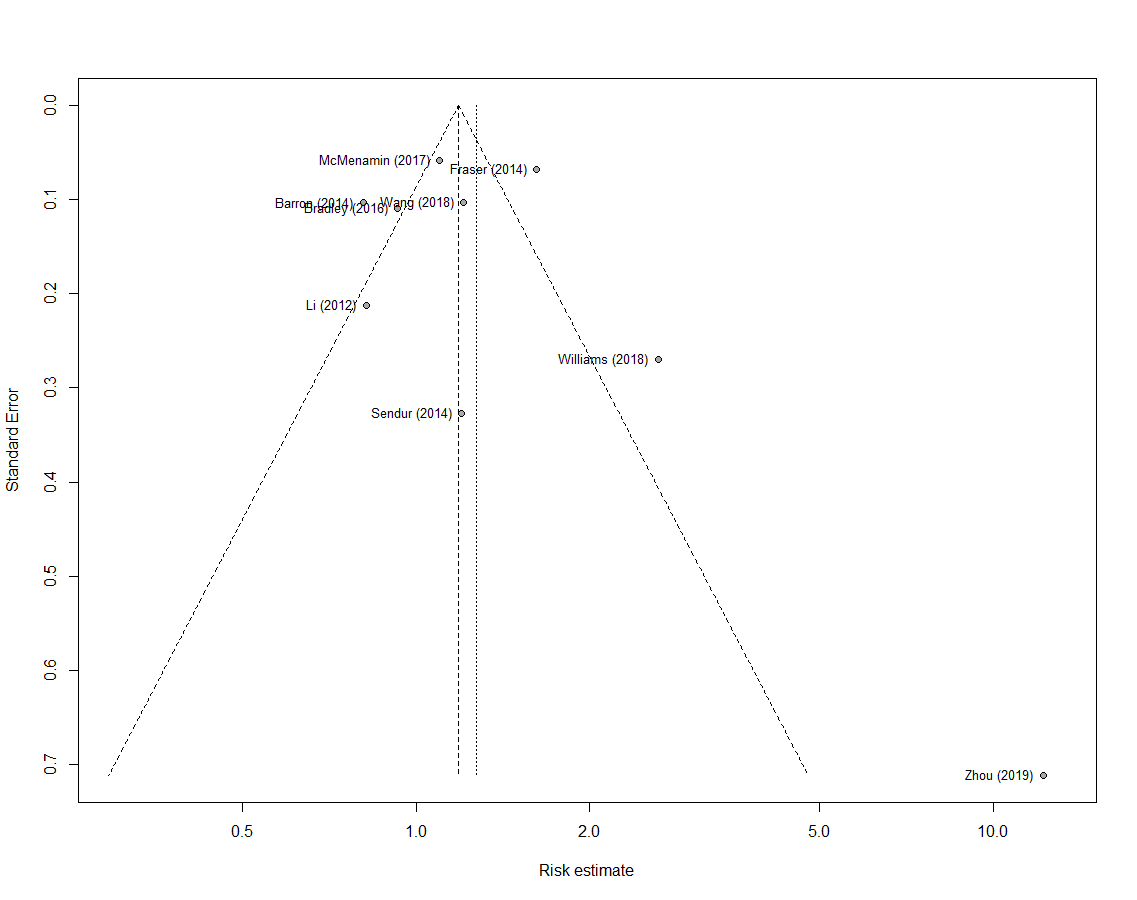
***

***Supplementary Figure 5- Funnel plot of studies included in the analysis of pre-diagnostic aspirin use and cancer recurrence***


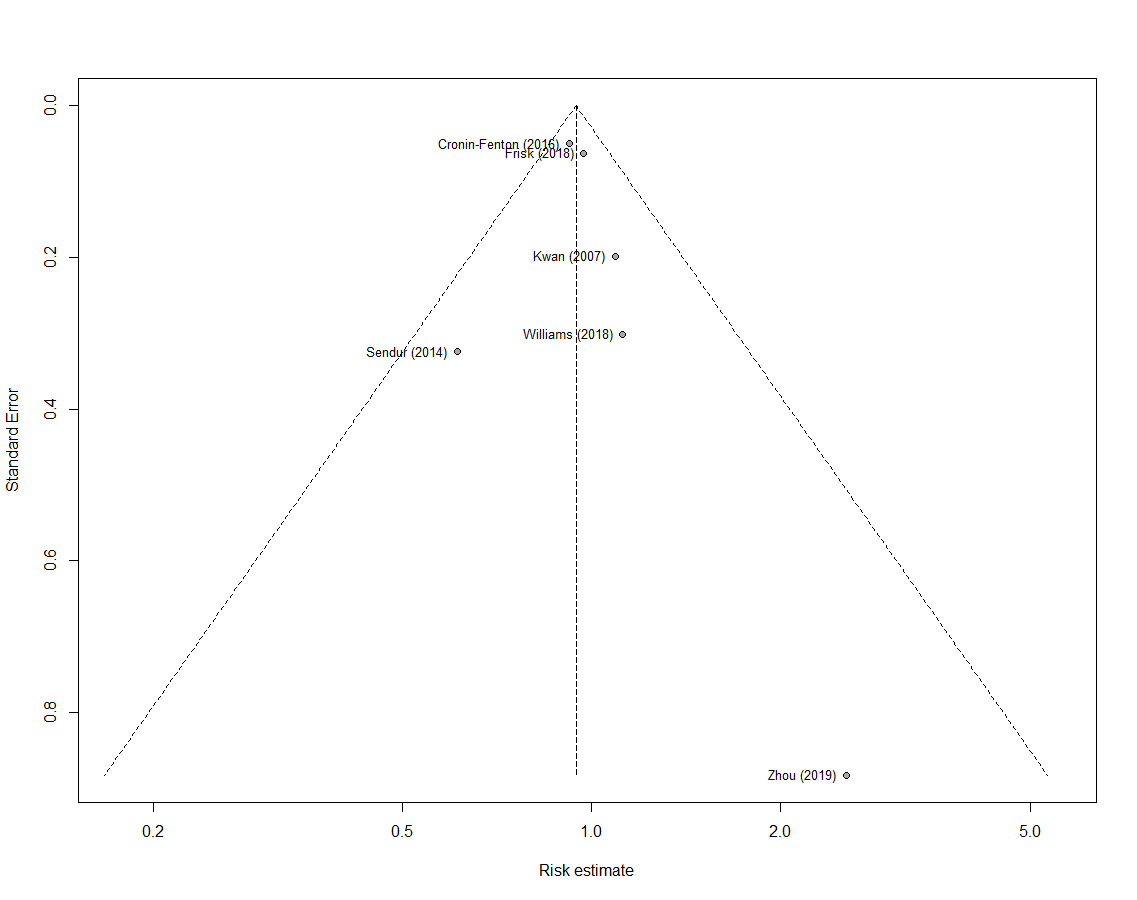


***Supplementary Figure 6a- Forest plot showing association between post-diagnostic aspirin use and breast cancer-specific mortality in patients with Stage I cancer at diagnosis***

***
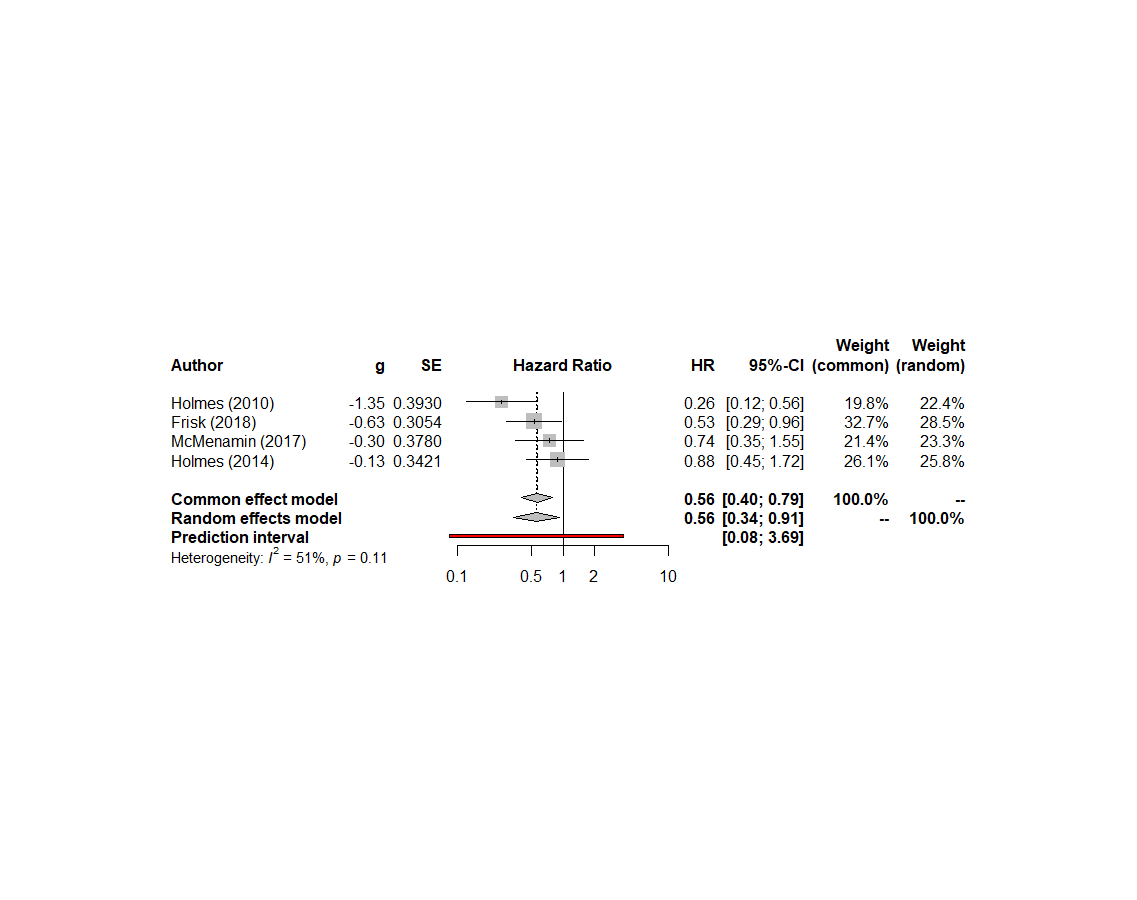
***

***Supplementary Figure 6b- Forest plot showing association between post-diagnostic aspirin use and breast cancer-specific mortality in patients with Stage II cancer at diagnosis***

***
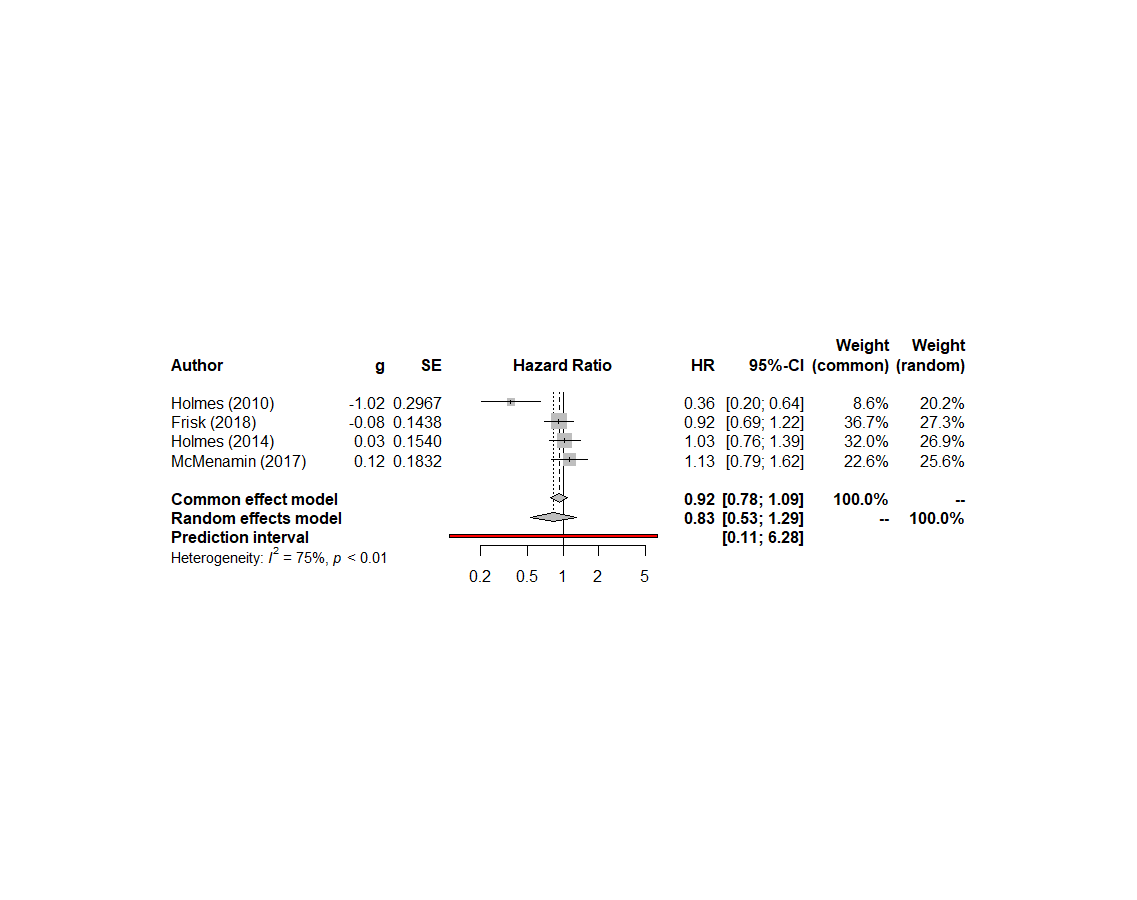
***

***Supplementary Figure 6c- Forest plot showing association between post-diagnostic aspirin use and breast cancer-specific mortality in patients with Stage III cancer at diagnosis***

***
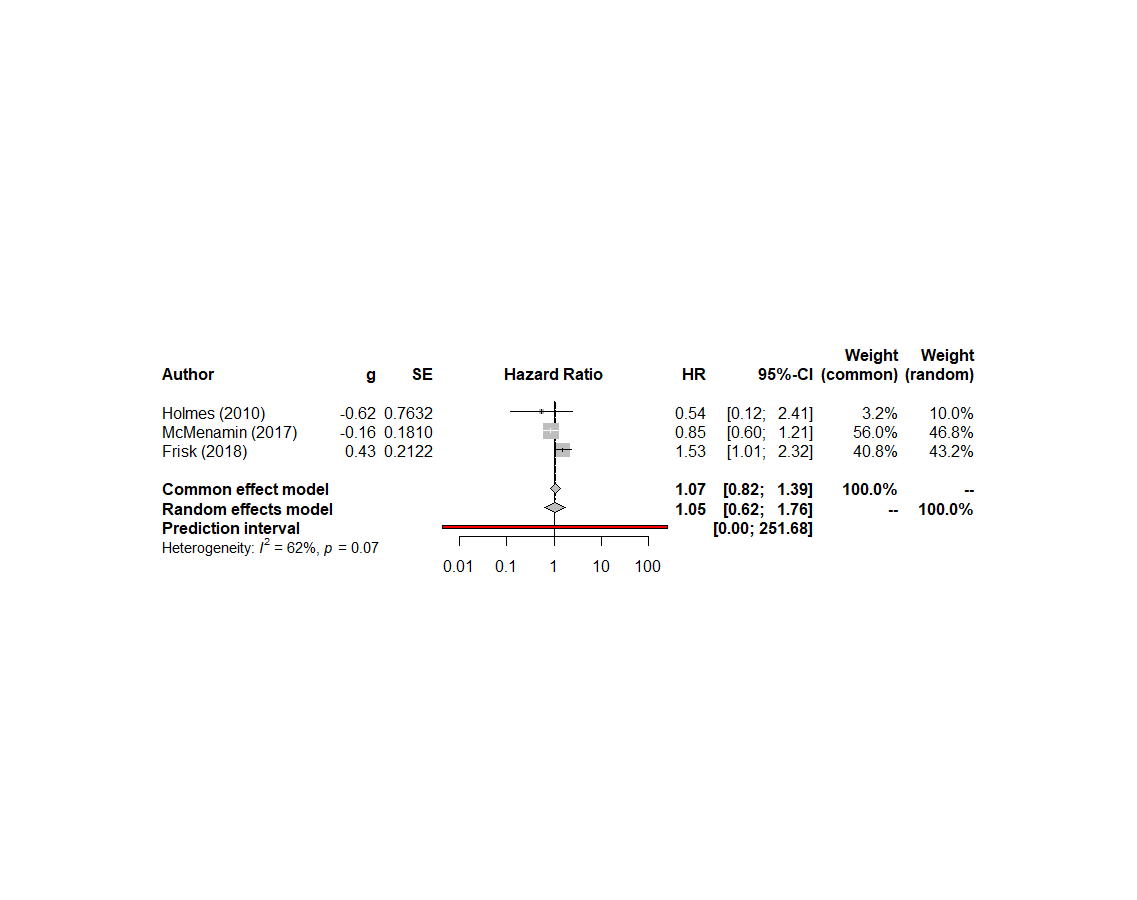
***

***Supplementary Figure 6d- Forest plot showing association between post-diagnostic aspirin use and breast cancer-specific mortality in patients with ER positive cancer at diagnosis***

***
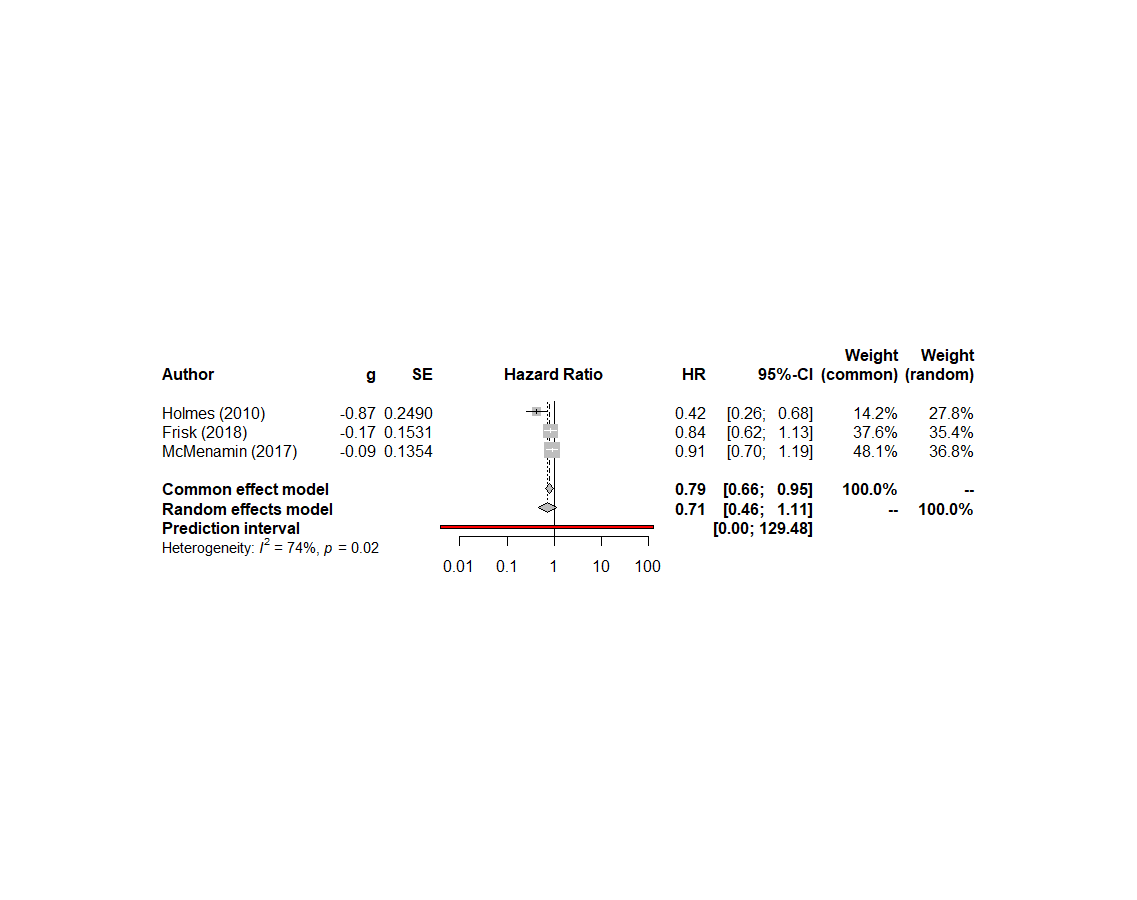
***

***Supplementary Figure 7- Bubble plot comparing association between post-diagnostic aspirin and breast cancer-specific death and proportion of each cohort who had node-positive cancer at diagnosis***

***
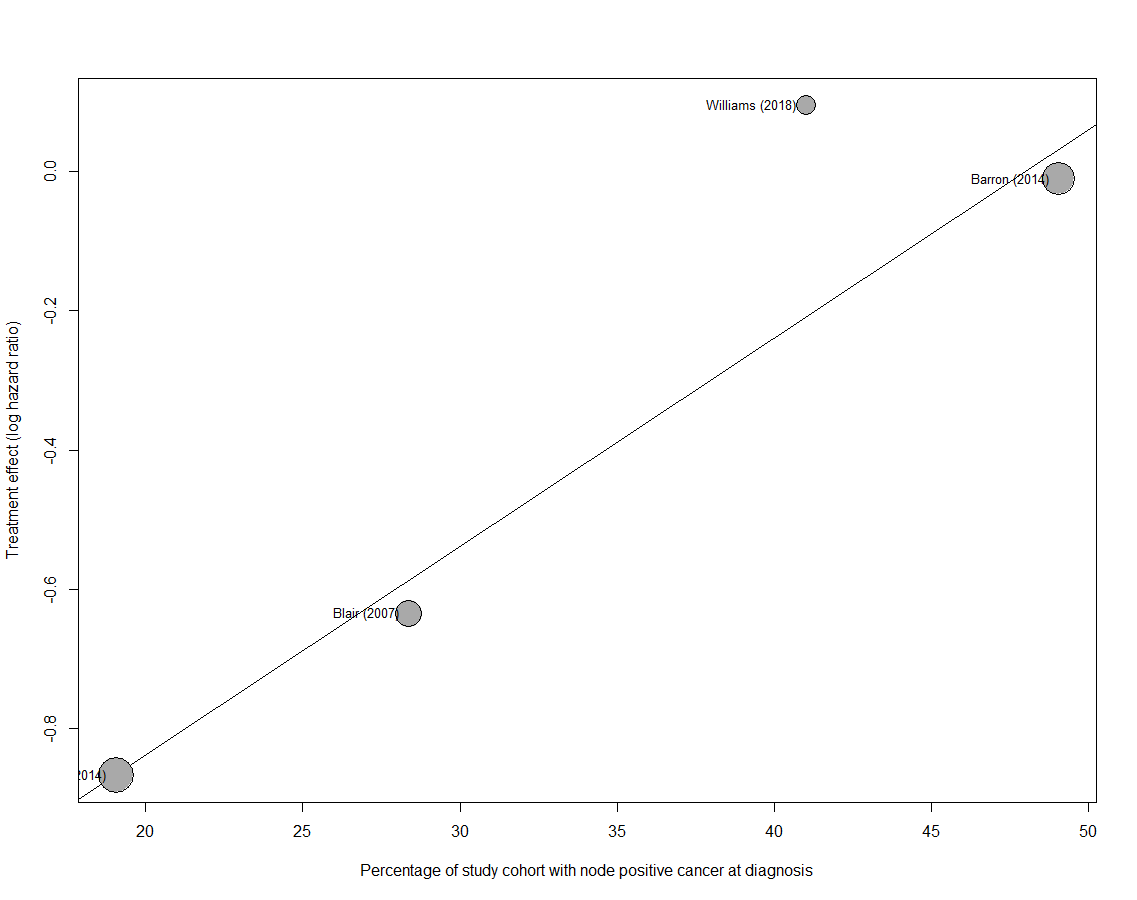
***

Fraser 2014

***Supplementary Figure 8- Funnel plot of studies included in the analysis of post-diagnostic aspirin use and breast cancer-specific mortality***

***
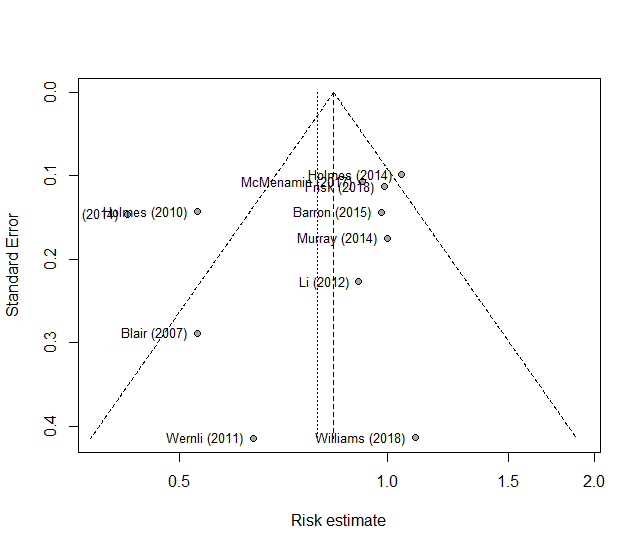
***

Fraser 2014

***Supplementary Figure 9- Bubble plot comparing association between post-diagnostic aspirin and all-cause death and proportion of each cohort who were Black***


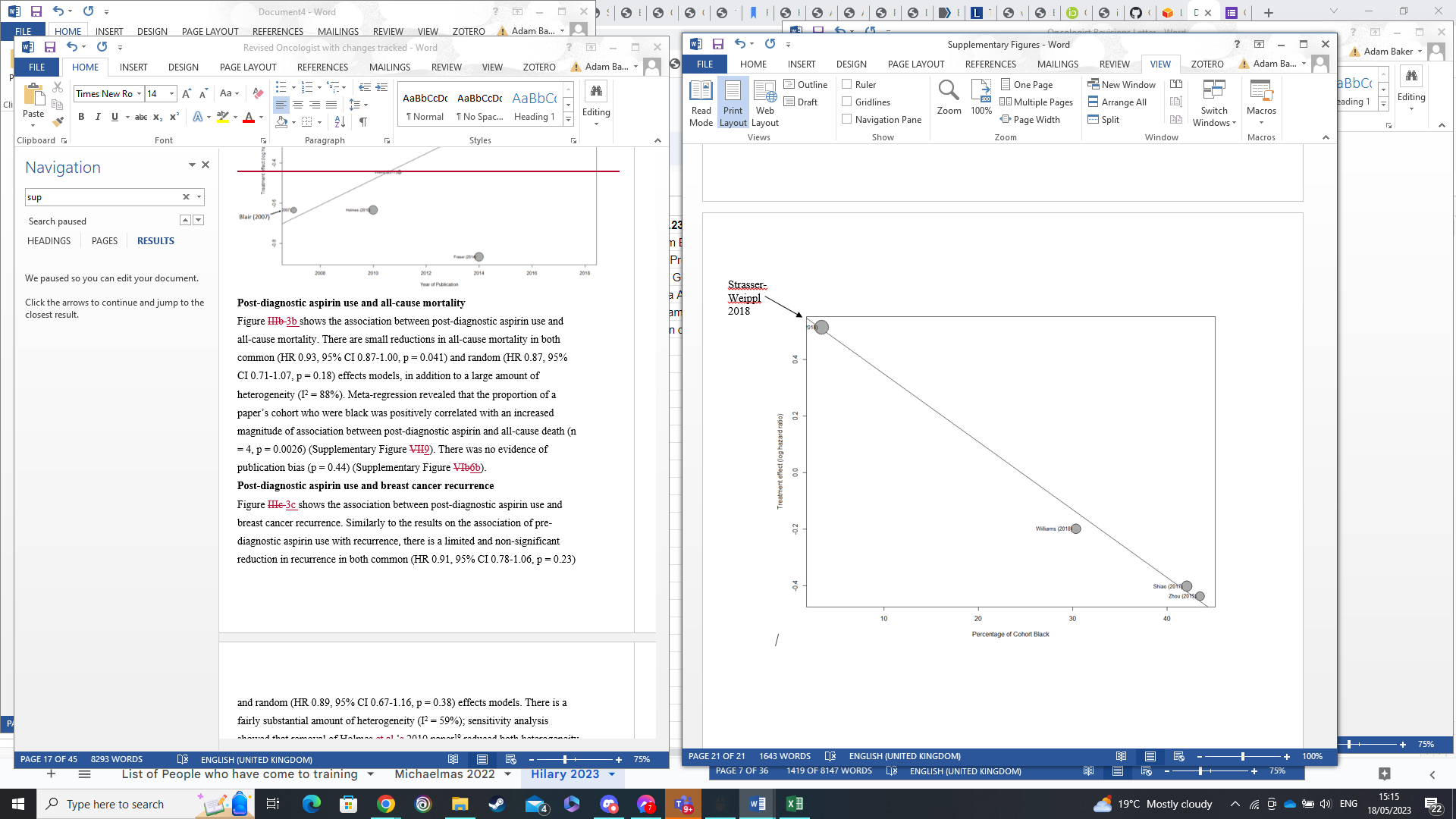


***Supplementary Figure 10- Funnel plot of studies included in the analysis of post-diagnostic aspirin use and all-cause mortality***

***
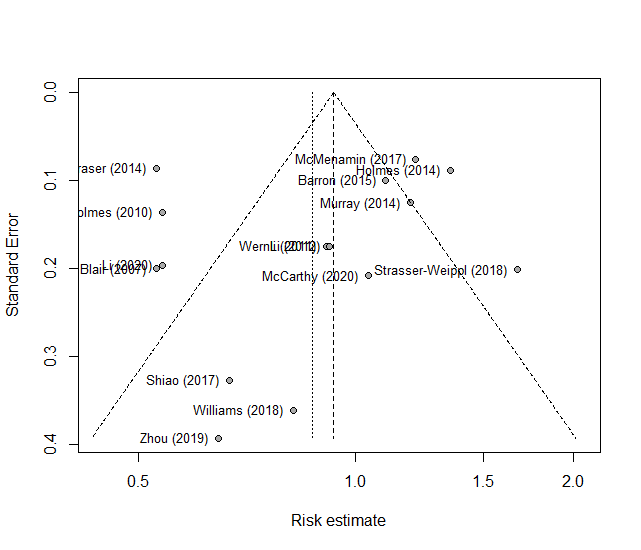
***

***Supplementary Figure 11- Funnel plot of studies included in the analysis of post-diagnostic aspirin use and cancer recurrence***


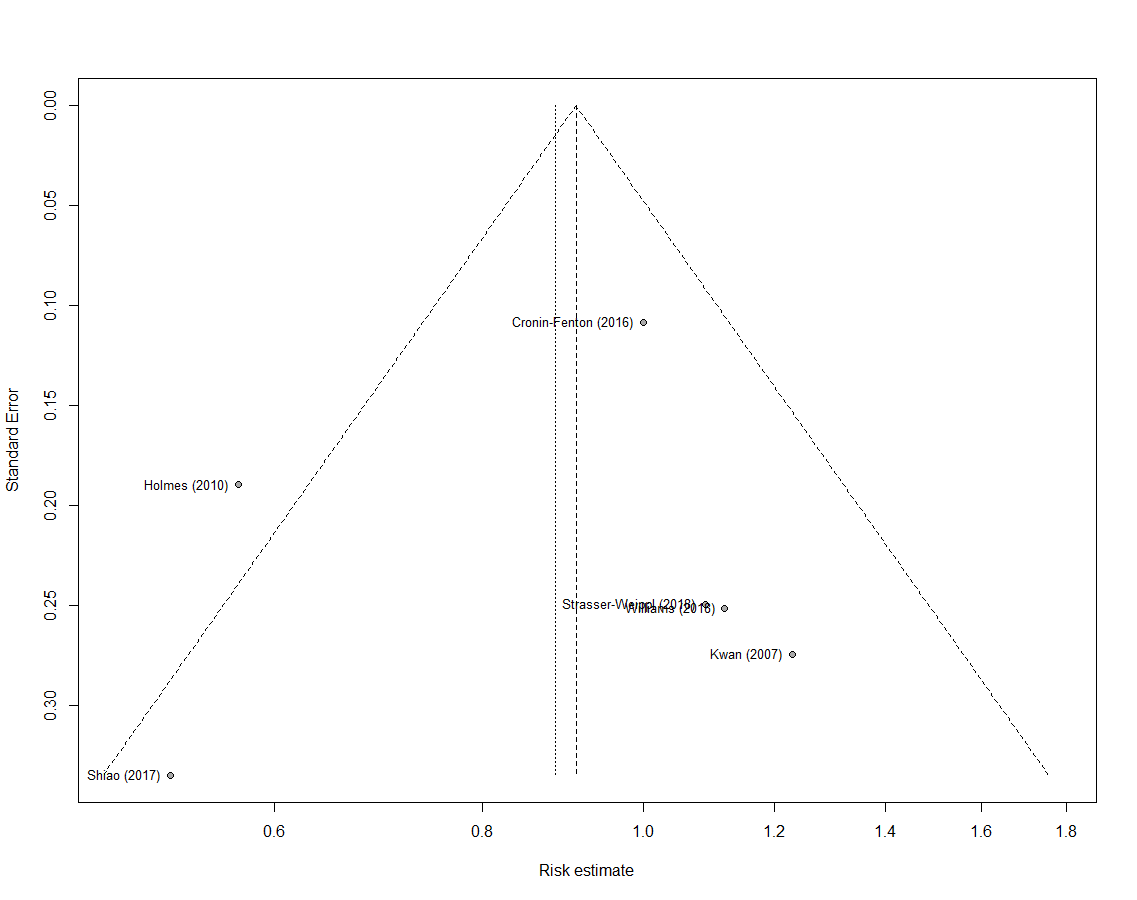

Supplement: oyad186_suppl_Supplementary_Figures_1-11 [file oyad186_suppl_supplementary_figures_1-11.docx]
