# Supplementary material for: Aspirin Use and Survival Among Patients With Breast Cancer: A Systematic Review and Meta-Analysis
Source: Oncologist. 2023 Jun 26;29(1):e1–e14. doi: 10.1093/oncolo/oyad186 (PMC10769789; doi:10.1093/oncolo/oyad186)
Supplement: oyad186_suppl_Supplementary_Tables_2-4 [file oyad186_suppl_supplementary_tables_2-4.docx]

***Supplementary Table 2- Confounders assessed in each study***

| Citation details | Confounders |
| --- | --- |
| Blair (2007) | Age, oestrogen receptor status, comorbidities (latter for all-cause mortality only) |
| Kwan (2007) | Age at diagnosis, race, cancer stage, tamoxifen treatment,  chemotherapy use, BMI, COX-2 inhibitor use |
| Holmes (2010) | Age, calendar year, smoking status, BMI, age at first birth and parity,  oral contraceptive use, menopausal status and use of hormone replacement, disease stage, treatment (hormonal, chemotherapy, radiation), protein and energy intake, physical activity, and weight change. |
| Wernli (2011) | Model A: Age at diagnosis, tumour stage, treatment. Model B: All of the above plus education, cigarette smoking, alcohol consumption,  body mass index, menopausal status, and family history of breast cancer ascertained at diagnosis. |
| Rothwell (2012) | N/A |
| Li (2012) | Age at diagnosis, race, education, BMI, menopausal status,  stage of breast cancer at diagnosis, ER/PR status, use of other NSAIDs. |
| Fraser (2014) | Age, socioeconomic status, TNM stage, tumour grade, oestrogen receptor status, surgery, radiotherapy, chemotherapy, adjuvant endocrine therapy, pre-diagnostic aspirin use. |
| Barron (2014) | In analysis of RR of aspirin use and lymph node-positive cancer: age, tumour size, tumour grade, comorbidity score, screen-detected tumour In analysis of aspirin and mortality: Age, tumour stage, tumour grade,  oestrogen receptor status, progesterone receptor status, HER2 status, comorbidity score. In analysis of post-diagnostic aspirin use, also adjusted for pre-diagnostic aspirin use |
| Barron (2015) | Smoking status, comorbidity score, tumour stage, tumour grade,  oestrogen receptor status, progesterone receptor status, HER2 receptor status, chemotherapy in year post-diagnosis, anti-oestrogen therapy in year post-diagnosis, lipophilic stain, hydrophilic stain, bisphosphonate medication, NSAID use, antidiabetic medication use. |
| Cronin-Fenton (2016) | Age, menopause status, histological grade, estrogen receptor/endocrine therapy, stage, primary surgery type, chemotherapy, prevalent comorbidities, baseline HRT use, post-diagnosis use of simvastatin, ACE-inhibitors and beta-blockers. |
| Bradley (2016) | For main analysis (this model used unless otherwise stated): age at diagnosis, stage, comorbidities, oestrogen and progesterone receptor status, diabetes. For aspirin affecting lymph-node status subanalysis: Age at diagnosis, size of breast tumour, grade, days from randomisation to diagnosis, diabetes. |
| Shiao (2017) | Ki-67 status (only for overall survival), age (only for overall survival  and disease survival), tumour clinical stage, lymphovascular invasion  (latter two for all of overall survival, disease free survival and distant metastatic rate) |
| McMenamin (2017) | Sex, age, year of diagnosis, deprivation, grade, stage, surgery, radiotherapy, chemotherapy, aromatase inhibitor (as time varying covariate), tamoxifen (as time varying covariate), statin usage (as time varying covariate), comorbities (e.g. acute myocardial infarction, congestive heart failure, peripheral vascular disease, cerebral vascular accident, pulmonary disease, peptic ulcer, liver disease, diabetes, renal disease) prior to diagnosis, hormone replacement therapy use in year prior to diagnosis. |
| Strasser-Weippl (2018) | Lymph node status, adujvant chemotherapy, treatment arm (exemestane vs anastrozole, celecoxib vs placebo), age, race, ER/PR status, nodal stage, adjuvant radiotherapy. |
| Frisk (2018) | One model (A) adjusted for age at diagnosis, stage, year of diagnosis, region, educational level, comorbidity before diagnosis (including inflammatory diseases, heart disease, cerebrovascular disease, atherosclerotic disease, thromboembolic venous disease, hyperlipidaemia, hypertension, peptic ulcer, liver disease, asthma). Another model (B) adjusted for all of the variables in (A), as well as statin use, metformin use, NSAID use, oncological treatment  (neoadjuvant/adjuvant chemotherapy, radiotherapy, endocrine therapy or trastuzumab). Also adjusted for prediagnostic aspirin use in analyses of post-diagnostic use. |
| Wang (2018) | Adjusted for age at diagnosis, race, physical activity, BMI, smoking, comorbidities (including hypertension, high cholesterol, diabetes,  myocardial infarction, stroke, and any cancer other than breast), co-medication use (including oral contraceptive pills, hormone replacement treatment, medications for hypertension, high cholesterol, myocardial infarction, stroke, and diabetes, and use of other NSAIDs). |
| Williams (2018) | Race, BMI, tumour size, tumour grade, lymphovascular invasion, nodal involvement, extracapsular extension, hormone receptor status, HER2 status, triple negative breast cancer status, receipt of mastectomy |
| Zhou (2019) | Race, tumour grade, tumour stage, oestrogen receptor status,  progesterone receptor status, HER2 status, treatment (chemotherapy,  radiotherapy, hormonal therapy), age |
| Li (2020) | Age, sex, race, household income, primary payer, patient location,  hospital characteristics, smoking, obesity, hypertension, hyperlipidaemia, diabetes, chronic kidney disease, COPD, congestive heart failure, history of DVT or pulmonary embolism. |
| McCarthy (2020) | Age, stage, progesterone receptor status, grade, chemotherapy, PIK3CA mutation status |
| Loomans-Kropp (2021) | Age at diagnosis, randomisation group (intervention/control), race,  smoking status, history of heart attack, stroke, hypertension or diabetes. |
| Holmes (2014) | Age at diagnosis, calendar year at diagnosis, time since diagnosis,  highest attained education level (as a proxy for socioeconomic status),  comorbities (disorders associated with increased or decreased use of aspirin, e.g. cardiovascular disease or asthma respectively). |
| Sendur (2014) | N/A |
| Murray (2014) | Model A adjusts for chemotherapy within six months of diagnosis,  radiotherapy within six months, tamoxifen (post diagnosis, during exposure period), aromatase inhibitors (post diagnosis, during exposure period), comorbidities (pre-diagnosis or during exposure period, including myocardial infarction, cerebrovascular disease, congestive heart disease, chronic pulmonary disease, peripheral vascular disease, peptic ulcer disease and diabetes), other medication exposure (post diagnosis, during exposure period, including statins, beta-blockers, ACE inhibitors, ARBs and metfomin) and smoking (pre-diagnosis, with missing included as a category). Model B adjusts for all confounders in Model A, in addition to stage and grade, restricted to 574 cases and 2,268 controls with available data. |

***Supplementary Table 3- Quality assessment of included cohort studies***

*Columns are as follows:*

*TS = Total score*

*RoEC = Representativeness of exposed cohort*

*SoNEC = Selection of non-exposed cohort*

*AoE = Ascertainment of exposure*

*DONPSS = Demonstration that outcome of interest was not present at start of study*

*SCMIF = Study controls for most important factor (age)*

*SCAF = Study controls for additional factor*

*AoA = Assessment of outcome*

*LoF = Length of follow-up*

*AoF= Adequacy of follow-up*

| **Study** | **TS** | **RoEC** | **SoNEC** | **AoE** | **DONPSS** | **SCMIF** | **SCAF** | **AoA** | **LoF** | **AoF** |
| --- | --- | --- | --- | --- | --- | --- | --- | --- | --- | --- |
| Blair 2007 | **8** | 1 | 1 | 0 | 1 | 1 | 1 | 1 | 1 | 1 |
| Kwan 2007 | **7** | 0 | 1 | 0 | 1 | 1 | 1 | 1 | 1 | 1 |
| Holmes 2010 | **7** | 0 | 1 | 0 | 1 | 1 | 1 | 1 | 1 | 1 |
| Wernli 2011 | **8** | 1 | 1 | 0 | 1 | 1 | 1 | 1 | 1 | 1 |
| Rothwell 2012 | **6** | 0 | 1 | 1 | 1 | 0 | 0 | 1 | 1 | 1 |
| Li 2012 | **9** | 1 | 1 | 1 | 1 | 1 | 1 | 1 | 1 | 1 |
| Fraser 2014 | **9** | 1 | 1 | 1 | 1 | 1 | 1 | 1 | 1 | 1 |
| Barron 2014 | **9** | 1 | 1 | 1 | 1 | 1 | 1 | 1 | 1 | 1 |
| Barron 2015 | **9** | 1 | 1 | 1 | 1 | 1 | 1 | 1 | 1 | 1 |
| Cronin-Fenton 2016 | **9** | 1 | 1 | 1 | 1 | 1 | 1 | 1 | 1 | 1 |
| Bradley 2016 | **8** | 1 | 1 | 0 | 1 | 1 | 1 | 1 | 1 | 1 |
| Shiao 2017 | **8** | 0 | 1 | 1 | 1 | 1 | 1 | 1 | 1 | 1 |
| McMenamin 2017 | **9** | 1 | 1 | 1 | 1 | 1 | 1 | 1 | 1 | 1 |
| Strasser-Weippl 2018 | **7** | 0 | 1 | 0 | 1 | 1 | 1 | 1 | 1 | 1 |
| Frisk 2018 | **9** | 1 | 1 | 1 | 1 | 1 | 1 | 1 | 1 | 1 |
| Wang 2018 | **7** | 0 | 1 | 1 | 0 | 1 | 1 | 1 | 1 | 1 |
| Williams 2018 | **7** | 1 | 1 | 1 | 0 | 0 | 1 | 1 | 1 | 1 |
| Zhou 2019 | **7** | 1 | 1 | 0 | 1 | 1 | 1 | 0 | 1 | 1 |
| Li 2020 | **6** | 0 | 1 | 1 | 0 | 1 | 1 | 1 | 0 | 1 |
| McCarthy 2020 | **7** | 0 | 1 | 1 | 1 | 1 | 1 | 1 | 1 | 0 |
| Loomans-Kropp 2021 | **7** | 0 | 1 | 0 | 1 | 1 | 1 | 1 | 1 | 1 |

***Supplementary Table 4- Quality assessment of included case-control studies***

| **Study** | Holmes 2014 | Sendur 2014 | Murray 2014 |
| --- | --- | --- | --- |
| **Total score** | **9** | **5** | **9** |
| **Case definition adequate?** | 1 | 0 | 1 |
| **Representativeness of cases** | 1 | 1 | 1 |
| **Selection of controls** | 1 | 1 | 1 |
| **Definition of controls** | 1 | 0 | 1 |
| **Study controls for most important factor (age)** | 1 | 1 | 1 |
| **Study controls for additional factor** | 1 | 0 | 1 |
| **Ascertainment of exposure** | 1 | 0 | 1 |
| **Same ascertainment method for cases and controls** | 1 | 1 | 1 |
| **Non-response rate** | 1 | 1 | 1 |
